# Tautomerism and switching in 7-hydroxy-8-(azophenyl)quinoline and similar compounds

**DOI:** 10.3762/bjoc.21.105

**Published:** 2025-07-10

**Authors:** Lidia Zaharieva, Vera Deneva, Fadhil S Kamounah, Nikolay Vassilev, Ivan Angelov, Michael Pittelkow, Liudmil Antonov

**Affiliations:** 1 Institute of Electronics, Bulgarian Academy of Sciences, 1784 Sofia, Bulgaria; 2 Institute of Organic Chemistry with Centre of Phytochemistry, Bulgarian Academy of Sciences, 1113 Sofia, Bulgaria; 3 Department of Chemistry, University of Copenhagen, DK-2100 Copenhagen Ø, Denmark

**Keywords:** azo dyes, *E*/*Z* isomerization, DFT, NMR, photochemistry, proton transfer, tautomerism, UV–vis

## Abstract

Tautomerism in two new azo dyes, based on 7-hydroxyquinoline, has been considered from the viewpoint of the proton crane concept. Although 8-(phenyldiazenyl)quinolin-7-ol exists in solution as a mixture of azo and two hydrazone tautomers, as shown by the experimental and theoretical results, upon irradiation switching, based on long-range proton transfer, occurs in a limited extent. 8-(4-Hydroxy-1,2,3,5-tetrafluorophenyldiazenyl)quinolin-7-ol exists as a single enol (azo) tautomer and the reduced basicity of the azo group nitrogen atoms does not allow shift of the tautomeric state neither upon changing the solvent, nor upon irradiation. Possibilities for molecular design, allowing to improve the capacity of 7-hydroxy-8-(azophenyl)quinolines, are considered in terms of stabilization of the azo tautomer and making possible long range proton transfer to the quinolyl nitrogen atom.

## Introduction

Azo compounds have long been utilized as dyes in industries such as textiles, printing, and coloring agents for various materials, due to their excellent color fastness, high stability, and ease of synthesis. The azo functionality allows for a wide range of colors to be achieved by altering the substituents on the azo group, improving the color intensity, lightfastness, and wash resistance [1–4]. In addition to their conventional use, azodyes have unique optical properties, defined by the *E*/*Z* isomerization [5–6] and by the tautomeric proton exchange [3,7–11], when a OH or NH group is present on a suitable position in the molecule. Both processes are strongly influenced by the structural variations and the environment (temperature, solvent properties, acidity and presence of other molecules).

The *E*/*Z* isomerization of the azodyes, caused by light irradiation [12–14] or electrochemically [15–16], has paved the way for the development of innovative materials and systems, including gels [17], metal-organic frameworks [18–21], photocatalysts [22–23], container molecules [24–26], drug delivery systems [27–29], photoresponsive polymers [30–37], photoswitches [12–13,38–41], optical storage devices [42–45] and systems for energy storage [46–47]. The phototautomerism of azodyes refers to the reversible isomerization process that occurs upon exposure to light, leading to exchange of a proton [48–49]. The obtained tautomeric forms have different optical and chemical properties, which make these molecules good candidates for molecular switches [50].

Recently, we have developed a series of 7-hydroxyquinoline Schiff bases, where the tautomeric proton transfer causes intramolecular twisting upon irradiation [51]. The process happens in the excited state and the competition between proton transfer and *E*/*Z* isomerization around the C=N bond leads to reduced efficiency of the tautomeric based switching [52].

**Scheme 1:**
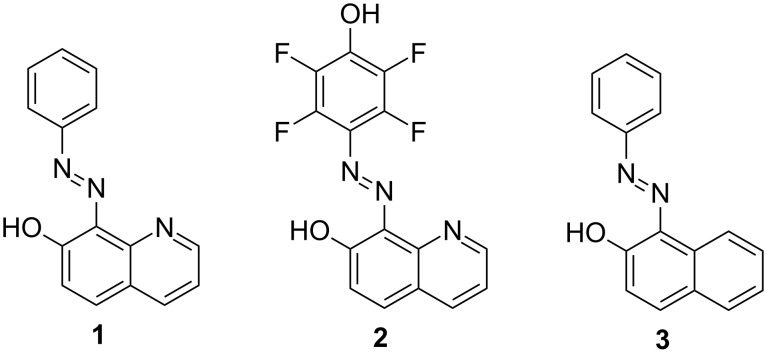
Investigated compounds.

In this respect, based on the excellent stability of the azo compounds, it is interesting to understand the effect of the competitive proton transfer and *E*/*Z* switching by replacement of the azomethine group with an azo group. Therefore, two new compounds, based on 7-hydroxyquinoline (**7OHQ**), namely (*E*)-8-(phenyldiazenyl)quinolin-7-ol (**1**, Scheme 1) and 8-(4-Hydroxy-1,2,3,5-tetrafluorophenyldiazenyl)quinolin-7-ol (**2**) were synthesized and studied in variety of solvents. The implemented additional OH functionality in compound **2** gives possibility to influence the tautomerism in this compound dynamically by protonation/deprotonation. The tautomeric and switching properties of compounds **1** and **2** have been revealed by using quantum-chemical calculations, UV–vis and NMR spectroscopy and UV irradiation. Compound **3**, whose tautomeric properties are well known and studied [11,53–55] is used as a model compound in which only short range proton transfer is possible.

## Results and Discussion

### Tautomerism in the ground state

**Scheme 2:**
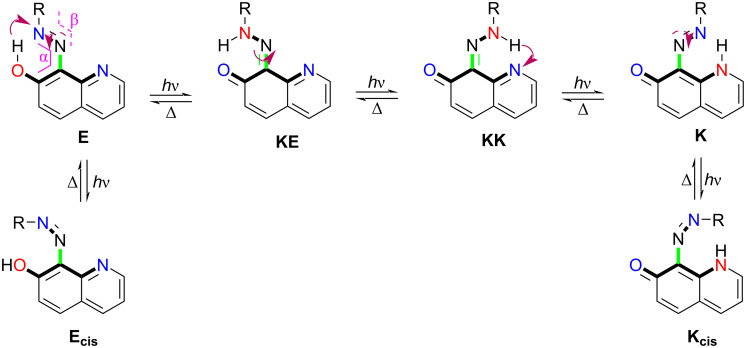
Long-range PT in the studied compounds along with undesired processes of *E*/*Z* isomerization. The individual steps are shown by violet arrows (with solid lines for the PT and with dash lines for the *E*/*Z* isomerization). The proton-donor and proton-acceptor sites are given in red and blue, respectively. The axle of intramolecular rotation is given in green. The twisting angles α and β are indicated in magenta.

As shown in Scheme 2, the studied compounds possess multiple tautomeric forms, allowing long-range proton transfer starting from the enol, azo, (**E**) and finishing to the end keto form **K**. As described in similar compounds, ideally for switching, the process begins with excitation of **E**, which, in the excited state, exhibits excited-state proton transfer (PT) to KE*. This changes the nature of the axle from single to double bond and leads to twisting, where conical intersection region is reached with the protonated crane (HN–N bond) and the deprotonated tautomeric frame (**7OHQ**) being almost perpendicular to each other. Then, the system relaxes to the ground state, populating both **KE** and **KK**, which undergo ground-state PT to **E** and **K**, respectively. Therefore, it is crucial to have the intermediate keto tautomers **KE** and **KK** higher in energy in the ground state, comparing to the paired terminal **E** and **K**, respectively, in order to provide efficient switching. The additional factors that are important include relatively low PT barriers (**TS**(**E-KE**) and **TS**(**K-KK**)) and relatively large twisting barrier (**TS**(**KE-KK**)). The former provides fast PT to the corresponding terminal states, while the latter allows accumulation of the **KK** and **K** as a result of the irradiation. When the crane part is flexible, the excitation of **E** can lead to *E*/*Z* isomerization, which competes with the initial excited-state PT process, reducing its efficiency [52].

The theoretical data, collected in Table 1, can shed light on the potential energy landscape in the ground state for the studied compounds. According to them neither **1** nor **2** is suitable for switching from **E** to **K** for couple of reasons. The most important one is the higher relative energy of **K** in respect of **KK**. This means that if **KK** is populated, according to the above described mechanism, the tautomeric proton cannot be released to **K**, i.e. the terminal form of the switching process is **KK**. This is also a kind of switching, which could be detected, because the **TS**(**KE-KK**) is large enough to allow accumulation of the switched form **KK**. As will be discussed in the next paragraph, in the case of compound **1**, the enol form is not the only existing tautomer, which means that the switching would not be clean.

**Table 1:** Relative stability (M06-2X/TZVP) and spectral characteristics of the ground-state tautomers of the studied compounds as well as relative energies of the transition states between them in toluene and in acetonitrile (in parentheses).

Structure /dipole moment [D]	Δ*E* [kcal/mol]	Δ*G*^0^ [kcal/mol]	UV–vis^a^ (B3LYP/TZVP// M06-2X/TZVP)		^1^H NMR [ppm]	
			λ_max_ [nm]	*f*		

**1E_cis_** 4.8	17 (16)	18 (17)	464 (467)	0.03 (0.04)	OH	3.97 (4.15)
**1TS**(**E-E_cis_**) 4.4	49 (49)	48 (47)				
**1E** 1.7	0.0 (0.28)	0.0 (0.0, *0.0*^b^)	398 397	0.67 (0.65)	OH	15.11 (15.21)
**1TS**(**E-KE**) 1.5	3.9 (3.9)	2.5 (2.1, *1.9*^b^)				
**1KE** 2.1	0.11 (0.0)	1.6 (1.2, *1.0^b^*)	432 (429)	0.64 (0.60)	NH	16.17 (16.05)
**1TS**(**KE-KK**) 9.3	41 (35)	42 (37, *37*^b^)				
**1KK** 7.8	1.3 (0.16)	2.4 (0.52, *0.53*^b^)	439 (436)	0.59 (0.57)	NH	15.53 (15.90)
**1TS**(**K-KK**) 9.1	9.8 (7.1)	7.7 (4.5, *4.5*^b^)				
**1K** 9.9	7.5 (3.8)	8.4 (4.4, *4.3*^b^)	456 (453)	0.02 (0.01)	NH	17.40 (16.98)
**1TS**(**K-K_cis_**) 11.1	54 (–)	54 (–)				
**1K_cis_** 11.2	27 (22)	28 (23)	515 (517)	(0.03) (0.04)	NH	7.58 (8.22)

**2E_cis_** 4.4	14 (14)	17 (16)	468 (470)	0.06 (0.06)	OH	4.07 (4.17)
**2TS**(**E-E_cis_**) 5.8	49 (49)	48 (48)				
**2E** 3.4	0.0 (0.0)	0.0 (0.0)	408 (406)	0.78 (0.74)	OH	14.06 (14.24)
**2TS**(**E-KE**) 3.7	5.0 (4.7)	3.5 (2.6)				
**2KE** 4.3	2.3 (1.8)	2.5 (2.4)	434 (434)	0.67 (0.65)	NH	16.12 (15.58)
**2TS**(**KE-KK**) 7.3	75 (42)	78 (42)				
**2KK** 6.2	2.7 (1.5)	4.0 (2.0)	438 (437)	0.62 (0.61)	NH	15.81 (16.20)
**2TS**(**K-KK**) 8.0	8.6 (6.3)	7.5 (4.3)				
**2K** 9.0	5.8 (2.2)	6.6 (2.9)	450 (453)	0.32 (0.01)	NH	16.74 (16.30)
**2TS**(**K-K_cis_**)	N/A (N/A)	N/A (N/A)				
**2K_cis_** 10.5	23 (N/A)	26 (N/A)	513 (N/A)	0.07 (N/A)	NH	7.93 (N/A)

c						
**3E_cis_** 3.2	18 (17)	20 (18)	498 (492)	0.06 (0.06)	OH	3.77 (3.96)
**3TS**(**E-E_cis_**) 2.2	48 (48)	47 (47)				
**3E** 1.3	0.0 (0.23)	0.0 (0.03)	432 (428)	0.55 (0.52)	OH	14.56 (14.64)
**3TS**(**E-KE**) 1.5	4.3 (4.3)	2.4 (1.3)				
**3KE** 1.8	0.25 (0.0)	1.1 (0.0)	450 (448)	0.59 (0.57)	NH	15.92 (15.73)
**3TS**(**KE-KK**) 11.1	47 (40)	48 (41)				
**3KK** 6.5	12 (9.8)	14 (11)	451 (448)	0.39 (0.42)	NH	9.71 (10.03)

^a^S_0_–S_1_ transitions, the simulated absorption spectra are shown as follows: **1** - in Figure 1 in toluene and in Supporting Information File 1, Figure S1 in acetonitrile; **2** – in Supporting Information File 1, Figure S2 in toluene; **3** – in Figure S3 in toluene; ^b^the values at 243 K are underlined; ^c^experimental Δ*G* values for the tautomeric equilibrium in **3** at room temperature [53]: 0.42 (cycloxehane), 0.34 (CCl_4_), −0.15 and 0.25 [56] (acetonitrile) kcal/mol.

According to relative energies and relative Gibb’s free energies, collected in Table 1, compound **1** should co-exist as a three component (**E**, **KE** and **KK**) tautomeric mixture in both toluene and acetonitrile. If the relative energies are taken into account in toluene **E** and **KE** are approximately equal as amount with a small presence (≈10%) of **KK**. The change of the solvent to acetonitrile does not change much the situation for the relatively equally polar **E** and **KE**, but leads to substantial stabilization of the more polar **KK**. The use of the relative free energies changes the ratios between these three tautomers, but does not change the overall situation in respect of the tautomerism. These theoretical results, obtained at the M06-2X DFT level, can be validated in several ways. The usual theoretical approach is to compare them to higher level of theory (either MPn or CCSD(T)) single point energies as shown in Table S1 (Supporting Information File 1). As seems from a comparison between the data from Table 1 and these in Table S1, the tautomeric fractions from **E** to **K**, obtained by MP4 correspond very well to the M06-2X relative energies from Table 1, while the relative Gibbs’ free energies are very near to the CCSD(T). Since this comparison does not answer the question about the reliability of the DFT data for **1**, it can be relied to a compound that is well-studied before. Compound **3** is a well-known tautomeric compound, a backbone of a large number of industrially used azo dyes [1,3] and therefore its tautomerism is studied in details. The existing experimental data for low polar solvents (cyclohexane and tetrachloromethane) indicate a Δ*G* value of around 0.4 kcal/mol [53] at room temperature, which gives a prevalence of the **E**-tautomer (a molar fraction roughly between 60 and 70%). The relative energy between **3E** and **3KE**, given in Table 1, is 0.25 kcal/mol (60% **E** and 40% **KE**) in toluene, which very well corresponds to the experiment bearing in mind that toluene is slightly more polar and could stabilize the more polar **KE** tautomer. The relative Gibbs’ free energies suggest a distribution of 88% **E** and 12% **KE**. The theoretical results for acetonitrile show that the tautomer fractions are almost equal, which reflects to the existing experimental Δ*G* values in acetonitrile, obtained by two different approaches (−0.15 [53] and 0.25 [56] kcal/mol).

**Figure 1:**
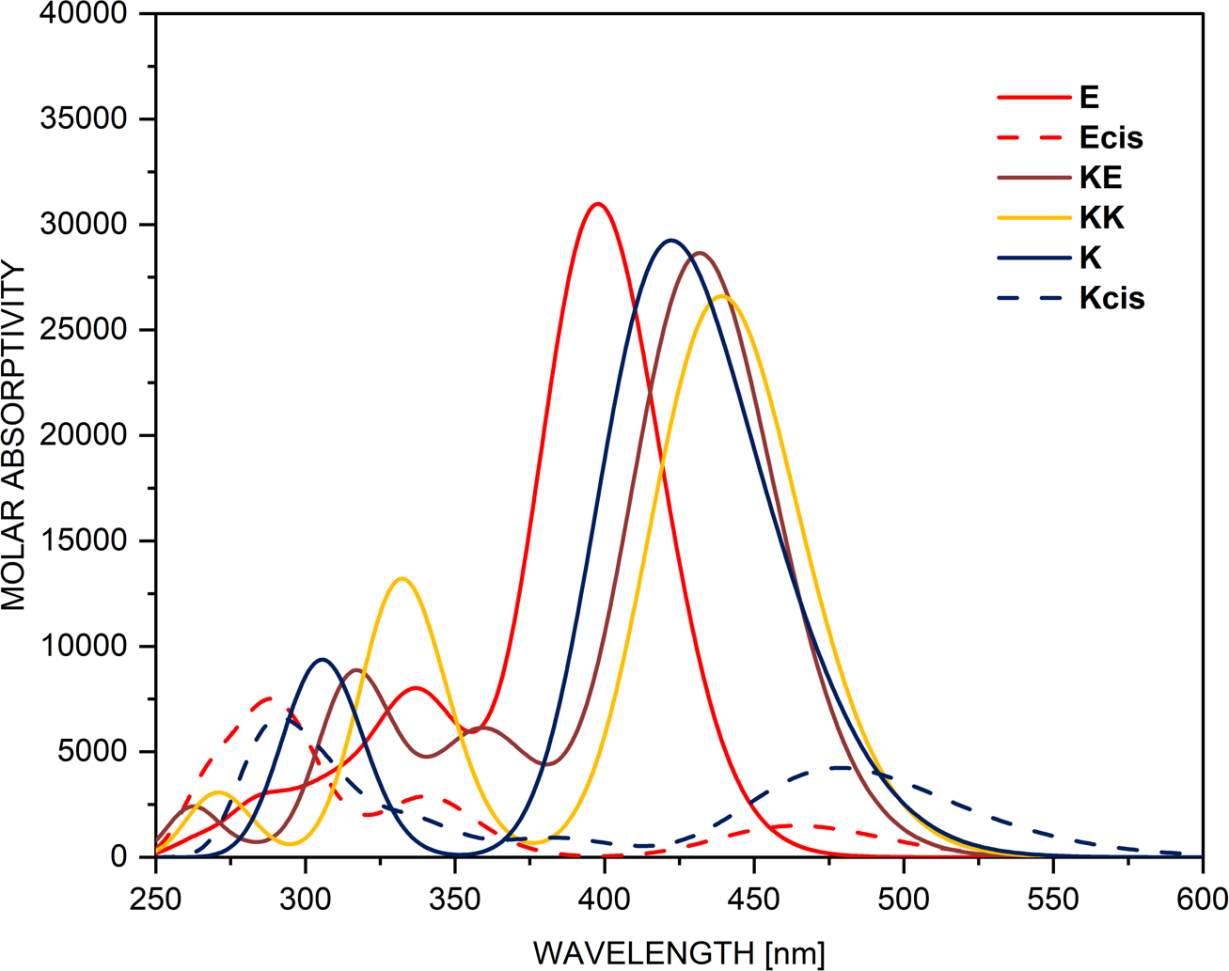
Simulated absorption spectra of the tautomers of **1** in toluene. The spectra in acetonitrile are shown in Figure S1 in Supporting Information File 1.

**Figure 2:**
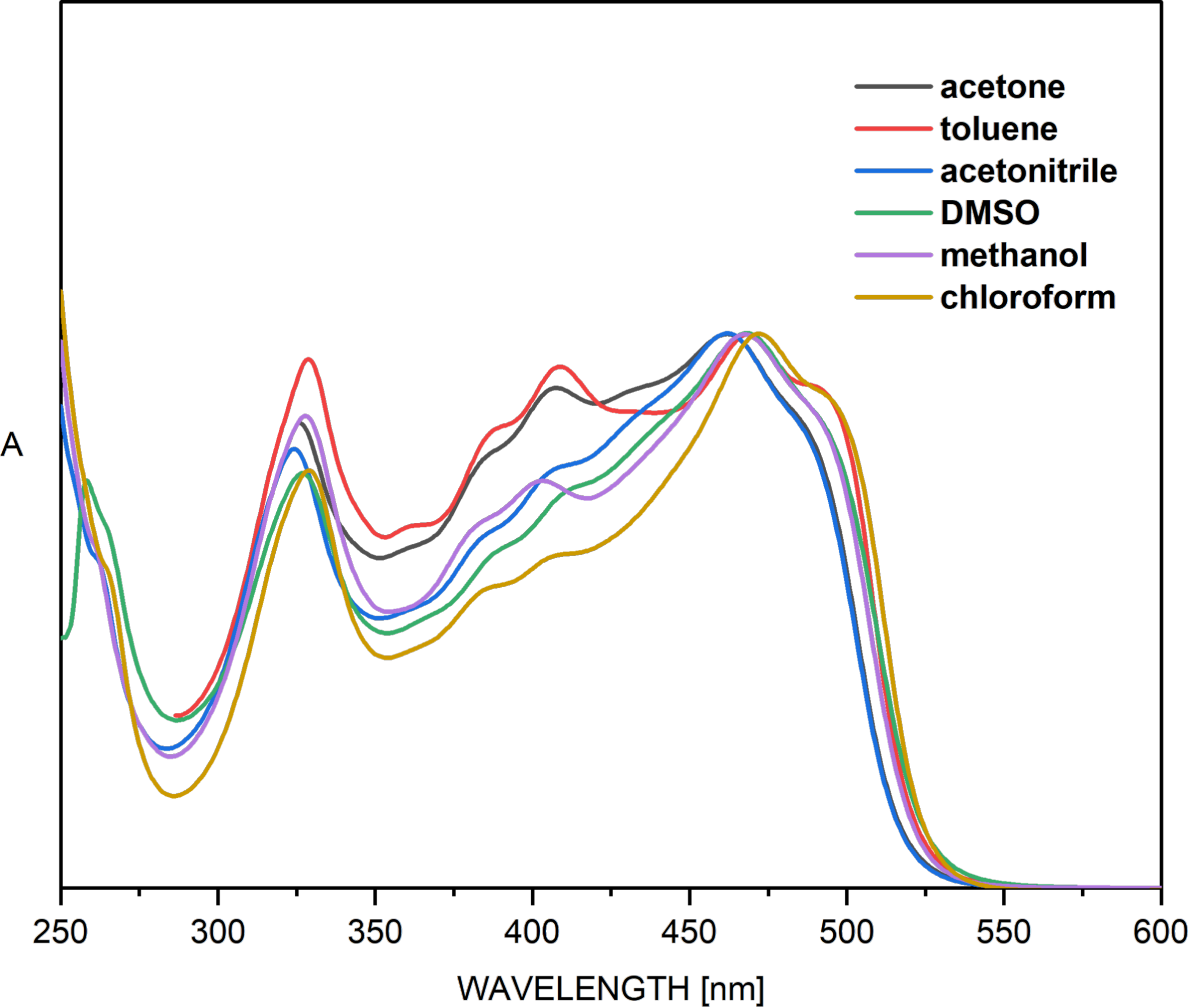
Normalized absorption spectra of **1**.

**Figure 3:**
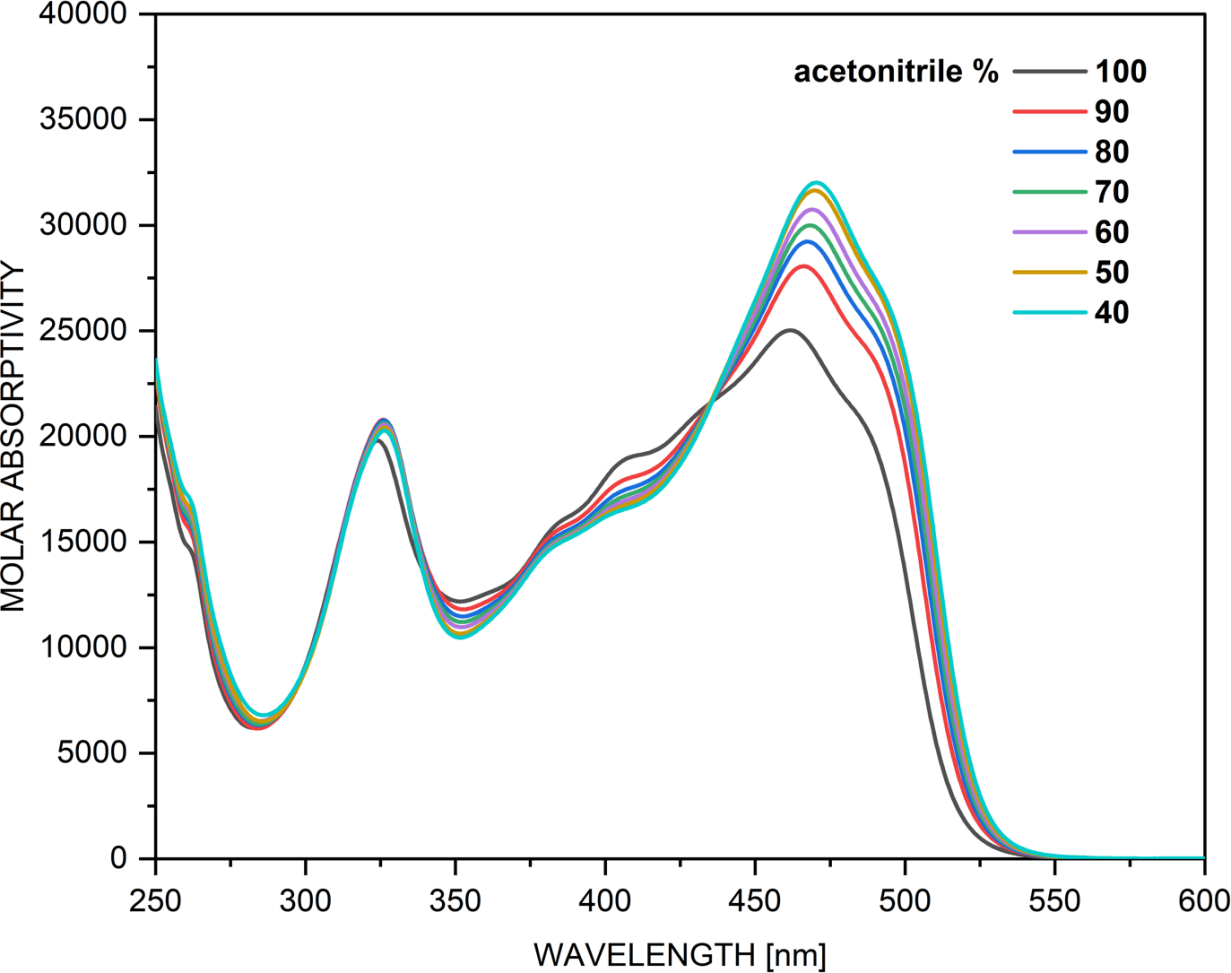
Absorption spectra of **1** in acetonitrile with stepwise addition of water.

Of course, the best way to validate the theoretical data for compound **1** is to study its tautomerism experimentally. The simulated absorption spectra of **1** are shown in Figure 1, while the experimental spectra in various solvents are given in Figure 2. It is obvious from the latter that solvent-influenced tautomerism takes place, but its interpretation is not a trivial work. The curves, shown in Figure 1, indicate why. It seems that the spectra of **KE** and **KK** are almost identical as position and intensity, which is reasonable, because they share the same chromophore system and, due to the stabilizing intramolecular hydrogen bonding, keep planarity. However, this situation means that changes in their molar fractions cannot be detected by use of UV–vis spectroscopy, i.e. the tautomerism could be considered only in the frame of change between **E** and (**KE**+**KK**). In the spectra, shown in Figure 2, two distinct spectral regions could be defined – around 400 nm, where the **E**-form absorbs (in analogy with the prediction from Figure 1 and the known individual spectra of the tautomers of **3** [55]) and around 450 nm, where the mixture of **KE** and **KK** absorbs. Bearing in mind that the predicted molar absorptivities of the tautomers are almost equal, it could be concluded that in toluene the ratio **E**/(**KE**+**KK**) is around 1, which corresponds very well to the relative energies from Table 1, predicting **E**/**KE**/**KK** as 52%/43%/5%. As seen from Figure 1 in all other solvents the content of (**KE**+**KK**) increases without disappearance of **E**. The reasons are different. While in acetonitrile this is the effect of the increased polarity of the solvent, leading to stabilization of the more polar keto tautomers, in the non-polar chloroform it is caused by its proton-donative nature (formation of a stabilizing intermolecular hydrogen bonding with the C=O group of the keto tautomers [51,56]). The effects of acetone, methanol and DMSO, as seen, are based only on the increased (comparing to toluene) dielectric constant of the solvent, stabilizing in different extent the more polar **KE** and **KK**. The further increase of the polarity of the environment by addition of water to acetonitrile (Figure 3) lead to increase of the content of the keto tautomers, but the enol form is still present.

**Figure 4:**
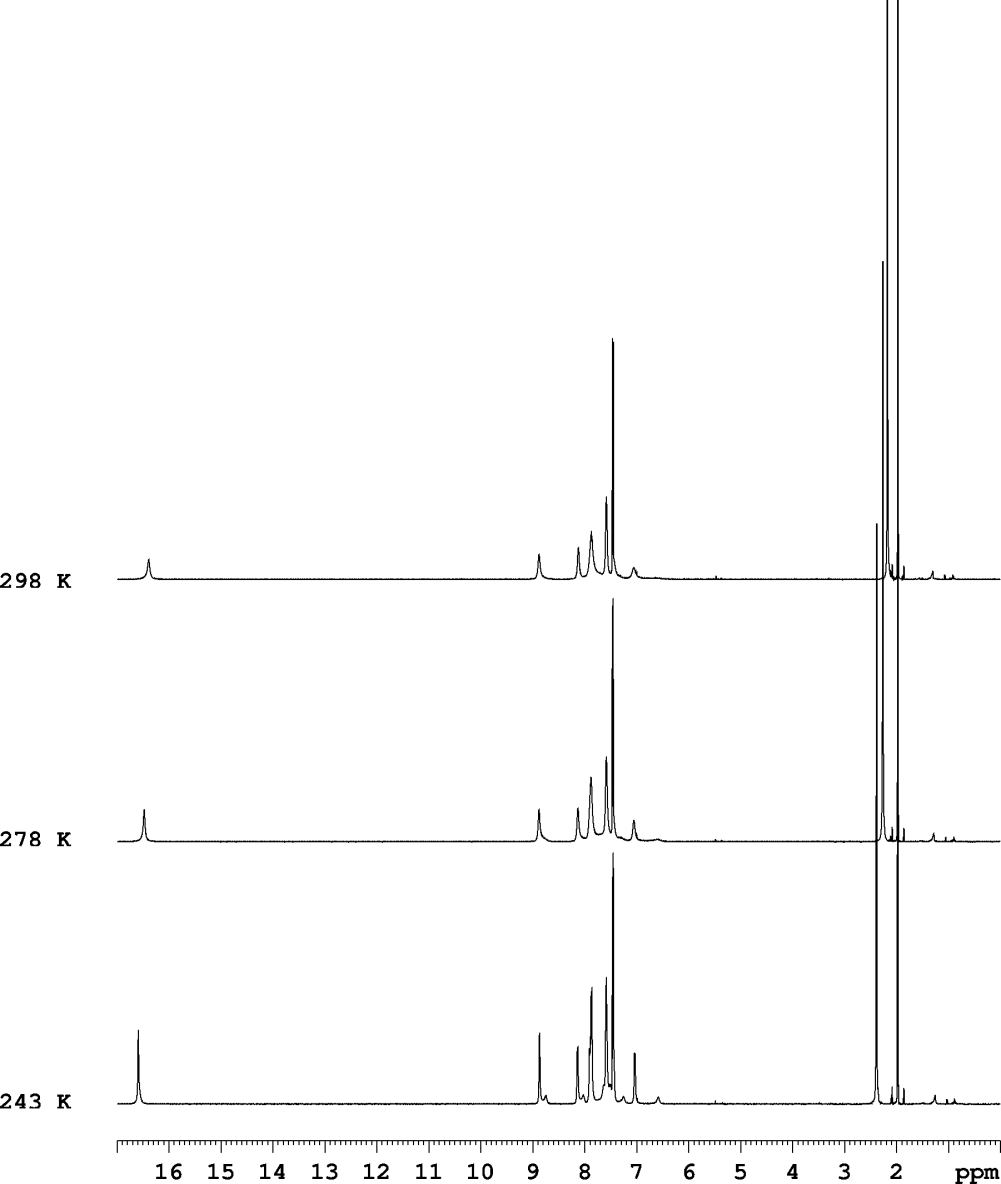
VT ^1^H NMR spectra of compound **1** in acetonitrile-*d*_3_.

As suggested by the theoretically predicted chemical shifts of the tautomeric proton (Table 1), the use of NMR could shed more light on the tautomeric composition in solution, again with some assumptions. The proton-transfer barrier between **E** and **KE** (**TS**(**E-KE**), Table 1) is almost negligible, which makes the process very fast for the NMR timescale. Consequently, the OH (in **E**) and NH (in **KE**) signals cannot be distinguished from each other, giving one average peak. This makes possible to analyze the tautomerism in **1** as a function of (**E**+**KE**) and **KK**. The NMR spectra of **1** in acetonitrile-*d*_3_ are in agreement with the above analysis of tautomeric equilibration based on theoretical calculations and UV spectra. At room temperature the proton signals are very broad and therefore the sample was cooled to 243 K (Figure 4). At this temperature the exchange process is slowing down and it is possible to register two exchanging protons (Figure S4 and Figure S5 in Supporting Information File 1). From COSY and NOESY spectra (Figure S6 and Figure S7 in Supporting Information File 1) most of the proton signals for the major and minor components can be assigned (see the note below Figure S4).

The calculated energy barrier of the process from **1E** to **1KE** in acetonitrile (2.11 kcal/mol, Table 1) is very low and this process should be fast in the NMR time scale. The cooling to 243 K does not change it much (Table 1, the italicized values). The calculated energy barrier of process from **1KE** to **1KK** in acetonitrile (37 kcal/mol in Table 1) is relatively high and this process should be slow in the NMR time scale. The conclusion from these calculations is that the major conformer is a result of fast exchange between **1E** and **1KE** and the minor conformer can be attributed to **1KK**. The measured carbon spectrum of **1** in CD_3_CN at 243 K (Figure S8 in Supporting Information File 1) supports this assignment as well. The HSQC spectrum (Figure S9 in Supporting Information File 1) helps in the assignment of the carbon spectrum.

The proton signal at 16.59 ppm seems to be a sum of two signals and a deconvolution with Lotenzian bandshape function was performed (Figure S5 in Supporting Information File 1). The obtained two signals have an integral intensity of 78 to 22, which is in agreement with other integrals in the proton spectrum. This means that at 243 K the mixture **1E**+**1KE** is still dominating, but the content of **1KK** slightly increases comparing to room temperature. This is not surprising bearing in mind that in the case of compound **3** the cooling in non-protic solvents (i.e. without the ability to form hydrogen bonding with the solute) shifts the equilibrium in direction of the keto tautomer [57].

**Figure 5:**
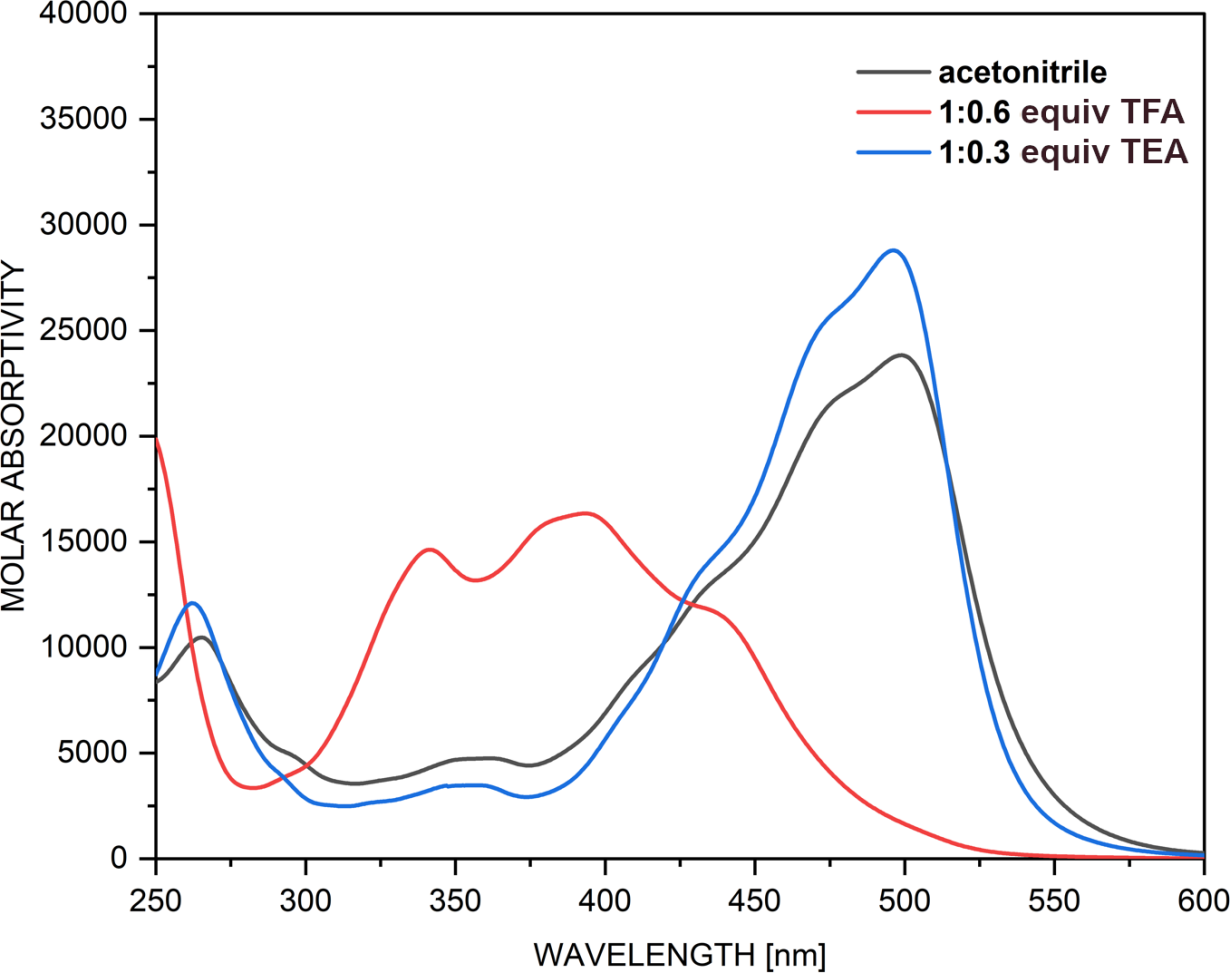
Changes in the absorption spectrum of **2** in acetonitrile upon addition of trifluoroacetic acid (TFA) and triethylamine (TEA). The addition of more TFA or TE does not cause further spectral changes.

According to the theoretical data, collected in Table 1, the enol form of **2** is substantially more stable and should predominantly exist in toluene. In acetonitrile some traces of **KE** and **KK** (both below 5%) could be observed in amounts around the detection limit of optical spectroscopy, which finally should not lead to substantial spectral changes comparing to toluene. The absorption spectra, shown in Figure S10 (Supporting Information File 1), suggest a different story. The spectra in toluene and CHCl_3_ are almost identical, with two intensive bands around 390 nm (assumed to **E** according to the simulated spectra, Figure S2) and 470 nm (assumed to **KE**/**KK**), which is rather surprising bearing in mind that chloroform, as a strong proton-donor solvent, always shifts the tautomeric equilibrium towards C=O-group-containing structures. In addition, the spectra in the remaining solvents show a single band around 500 nm independent on the nature of the solvent. Obviously, these data cannot be interpreted in the frame of the concept for a tautomeric equilibrium. The rotor in **2** contains a OH group surrounded by fluorine atoms, which could spontaneously deprotonate in polar solvents, while the tautomeric OH/NH group is always a part of strong intramolecular hydrogen bonding and the possibility for deprotonation is low. The deprotonation of the phenyl OH group is also supposed by the computational quantum chemistry calculations. As seen from Table S2 in Supporting Information File 1, the deprotonation of the tautomeric proton requests substantially more energy, while the deprotonation of the phenyl OH group leads to a single **E^−^** tautomer. This fact is confirmed by a simple experiment. As seen from Figure 5, in the spectrum of **2** in acetonitrile a single band is observed at 500 nm. Upon addition of a very small amount of trifluoroacetic acid this band disappears and a new band at 400 nm appears, suggesting that the red-shifted band can be attributed to **E^−^**. The spontaneous deprotonation in acetonitrile is not completed, because the addition of triethylamine leads to rise of the band at 500 nm. The hypothesis for deprotonation in acetonitrile is nicely confirmed by the simulated spectra of **E** and **E^−^**, shown in Figure S11 and Figure S12 in Supporting Information File 1. As seen the intensity of the **E^−^** is substantially larger, exactly as observed in Figure 5.

### Excited-state proton transfer and switching

The lack of emission in the azo dyes is attributed to the availability of non-bonding electron pairs at the nitrogen atoms from the azo group and the possibility for *cis*/*trans* isomerization upon excitation, which leads to conical intersection deactivation from the excited to the ground state. And while there is substantial number of experimental and theoretical (some of them at very high level of theory [58–60]) investigations concerning the *cis*/t*rans* isomerization in azobenzenes [61–74], the number of studies, dealing with the excited-state behavior of compounds where an OH group is situated on the ortho position in respect of the azo group, is very limited. It is not surprising because in these cases a possibility for ESIPT exists along with the *cis*/*trans* isomerization, creating a rather complicated interplay between two competitive processes. Experimental [49,75–80] and theoretical [81–84] studies of compound **3** and derivatives clearly indicate the excited-state competition between the ESIPT from enol to keto form, which leads to very weak emission, and the *trans* to *cis* isomerization, leading to emissionless deactivation to the ground state.

**Figure 6:**
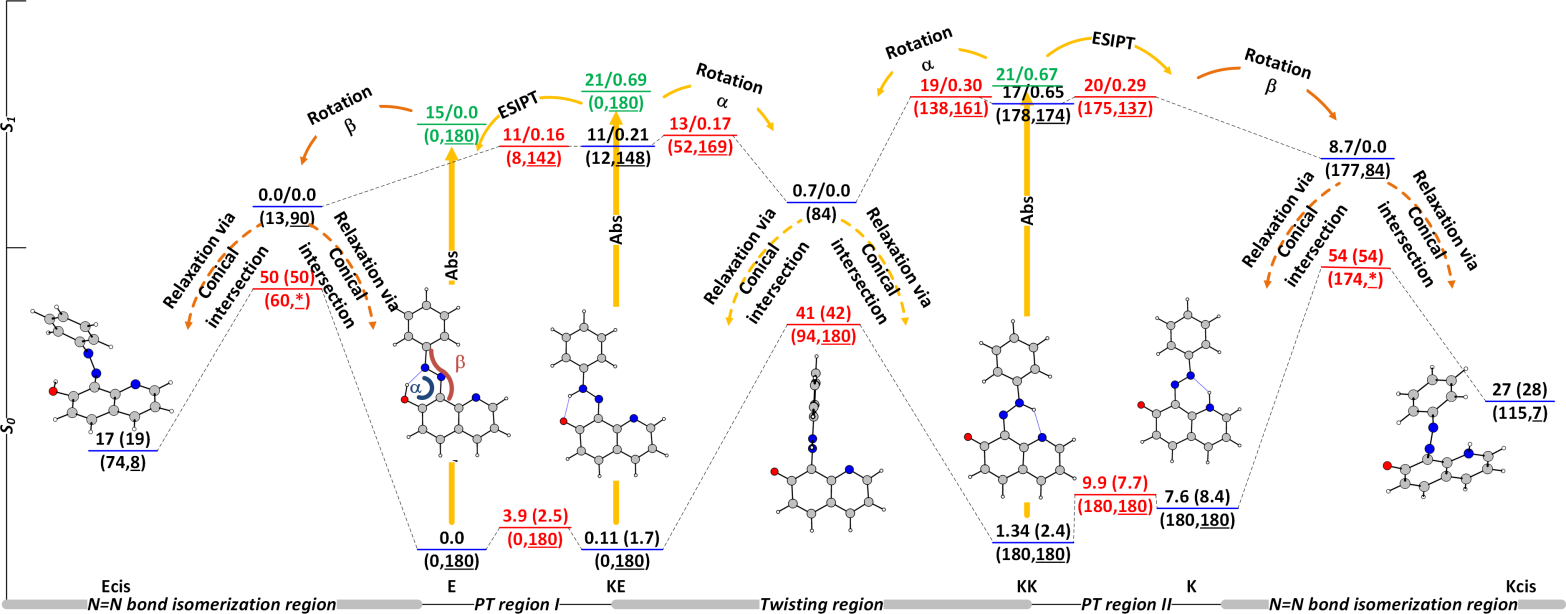
Ground (M06-2X/TZVP) and excited (CAM-B3LYP/TZVP) state potential energy surface of compound **1** in toluene presented as relative energies in kcal/mol. The values of the angles α and β (see also Scheme 2) are given in brackets (β is underlined). The asterisk indicates *trans* to *cis* isomerization via inversion mechanism [67]. In the excited state the relative energy is followed by the oscillator strength of the corresponding state. The Franck–Condon states of the tautomers, existing in solution, are given in green.

The potential energy surfaces (PESs) of **1** in the ground and excited state, sketched in Figure 6, show a very complicated picture. The ground-state tautomerism, discussed above, leads to a tautomeric mixture of **E**, **KE**, and **KK**, each of them being excited upon irradiation. As already shown [82], the first singlet excited state of **E** is dark. According to the results from Figure 6, E* can spontaneously go to the t*rans* to *cis* conical intersection (CI) via twisting ground the N=N bond (angle β) returning back to the ground state. Although from the spectral point of view (Figure 1) the obtainment of a mixture of **E** and **Ecis** should result in a decrease of intensity of the band around 400 nm, the same process in **3** is very fast to be recorded by conventional UV–vis spectroscopy, suggesting preferable population of intramolecular hydrogen bonding stabilized **E**. Comparing the angles α of the E* form (13°) and the transition state between **E** and **Ecis** (60°) the former is nearer to the geometry of **E**, suggesting preferable population. The excitation of **KE** gives three possible pathways: returning back to ground state by emitting, ESIPT to E* (and relaxation to the ground state by CI in the N=N bond isomerization region) and twisting around the double bond axle C=N (change of α) to the CI in the twisting region populating **KE** and **KK** in the ground state. No measurable emission of **1** was detected in any of the studied solvents, excluding the first pathway. The ESIPT process is practically barrierless leading to the CI in the N=N bond isomerization region with preferential population of **E** as discussed above. The third pathway could lead to population of **KK**, rise of its existing amount and, consequently, to rise of the absorption band at around 470 nm and restoration with the time of the equilibrium tautomeric state. No appearance of **K** is expected as a result of higher energy as discussed previously.

**Figure 7:**
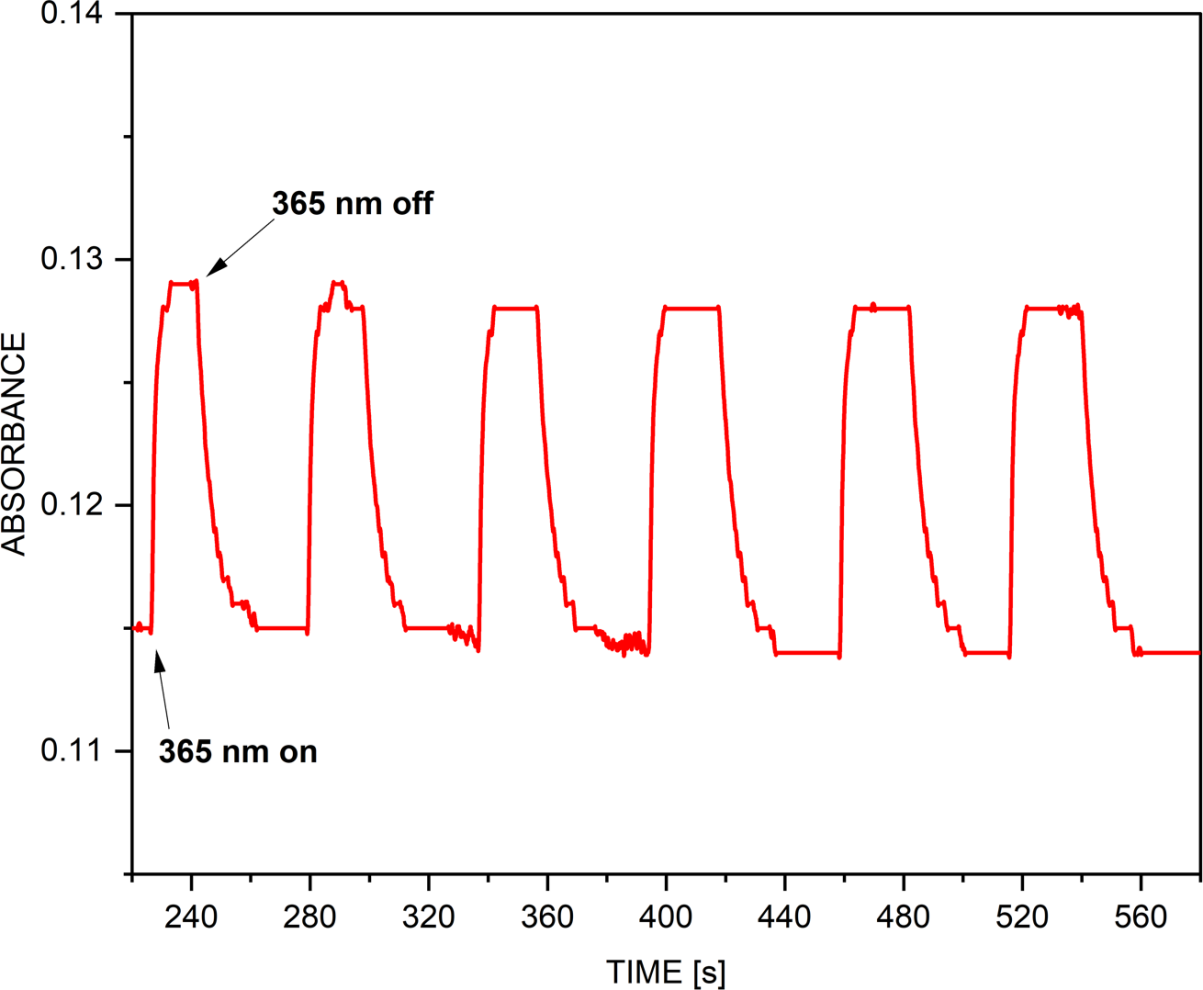
Changes of the absorbance of compound **1** at 465 nm in toluene upon turning on and off the irradiation source (365 nm).

Actually, the third process is shown in Figure 7. Upon irradiation at 365 nm a slight rise of the absorbance around 470 nm is observed. The process is fast (0.738 ± 0.029 s^−1^ for the forward transition from the equilibrium mixture **E**+**KE** to **KK** and 0.177 ± 0.031 s^−1^ for the transition back) and fully reversible, indicating that a switching between **E**+**KE** and **KK** occurs from the PT region I to the PT region II as indicated in Figure 6. The switching is observed in toluene, but not in acetonitrile, which is probably due to the substantial decrease of **TS(KE-KK)** relative energy (see Table 1) moving it to the shorter time window. The dipole moments of KE* and the surrounding transition states are approximately the same, which excludes changes in the stabilization going from toluene to acetonitrile. The results indicate that the excitation of **KE** leads preferably to return to the ground state via CI in the N=N bond isomerization region and, in a very limited extent, to switching to **KK**. According to the PESs in Figure 6, the excited **KK** behaves in a way similar to **KE** and the spectral changes cannot be distinguished from these of **KE** due to the similarities in the absorption spectra.

Compound **2** does not emit in the studied solvents and its absorption spectra in toluene do not show detectable changes upon irradiation. The twisting barrier (**2TS(KE-KK)** in Table 1) here is substantially larger comparing to **1**, which means that if switching to **KK** occurs the relaxation process back should be slower. The theoretical calculations give the possible reason for lack of switching – due to the low basicity of the proton-accepting nitrogen atom, the KE* form spontaneously goes to E*, which closes the channel to the twisting region. On the other side there is substantial number of papers describing the very long-lived *cis* isomers in *ortho*-halogen-substituted azobenzenes [38,85–88]. Obviously, in the case of compound **2** the relaxation back to the ground state through the CI in the N=N bond isomerization region, which is the only option in this compound to return back to the ground state, populates exclusively the ground state **E** instead of **Ecis**.

### Is there a potential for clean switching in **1**?

Although it was shown above that there is a switching from the mixture **E**+**KE** to **KK** in **1** in toluene, the compound does not fulfil the requirements for clean switching. Returning back to Scheme 2, the clean switching can be defined, in general terms, as transition from the pure **E** (or **KE**) to the pure **K** (or **KK**). In order to classify compound **1** as a real proton crane, the switching has to be from **E** to **K**.

As shown above, in the case of **1**, there is a mixture between **E**, **KE** and **KK** (problem with a clean off-state of switching) and the end tautomer **K** is higher in energy comparing to **KK** (problem with possibility to achieve a clean on-state of switching). All this means that structural changes are needed in order to find an azo compound, based on 7-hydroxyquinoline, where the individual tautomers have energies ranked in the following way: *E***_E_**<<*E***_KE_**, *E***_KK_** and *E***_K_**<*E***_KE_**, *E***_KK_**, *E***_E_**<*E***_K_**. In order to assure that in the initial solution (prior irradiation) only **E** is present, the relative energy of **K** should be at least 2 kcal/mol higher to be considered negligible from the viewpoint of optical spectroscopy. In other words, three conditions can be defined for clean switching:

- The relative energy between **K** and **E**, Δ*E*(**K**-**E**) > 2 kcal/mol, in order to have only pure **E** in solution;
- Δ*E*(**KE**-**E**) > 2 kcal/mol, again to assure presence only of **E**;
- Δ*E*(**KK**-**K**) > 2 kcal/mol in order to provide conditions for complete transfer from **KK** to **K** upon irradiation.

**Figure 8:**
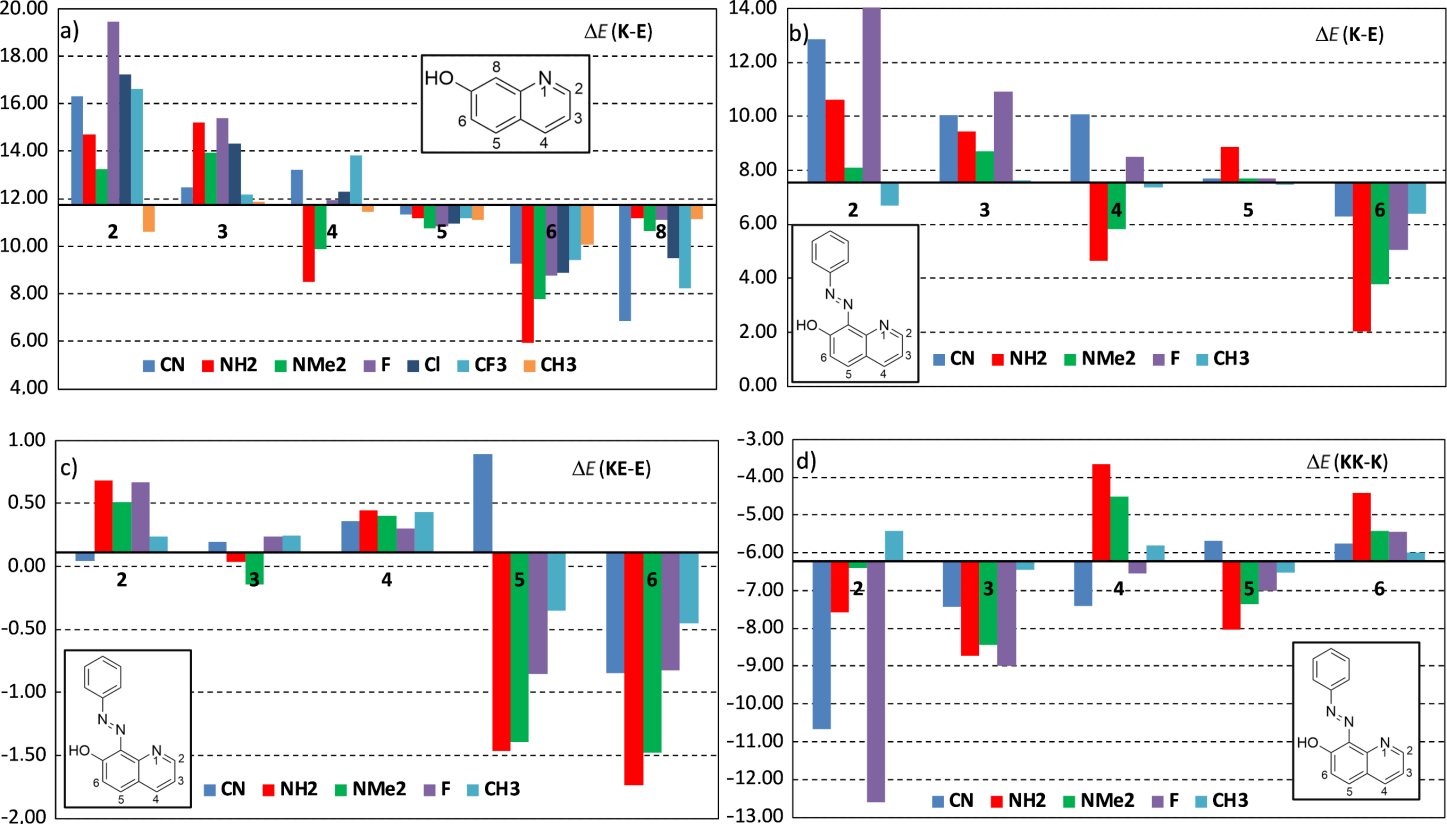
a) Change of Δ*E*(**K**-**E**) in kcal/mol as a function of the substitution on different positions (2–6) in **7OHQ** in respect of the value for the unsubstituted **7OHQ**; changes of Δ*E*(**K**-**E**) (b), Δ*E*(**KE**-**E**) (c), and Δ*E*(**KK**-**K**) (d), all in kcal/mol, in **1** as a function of the substitution at a different position (2–6) in the 7-hydroxyquinoline part in respect of the corresponding values for unsubstituted **1**. All results are for toluene.

The relative stabilization of **K** in respect of **E** depends mainly on the substitution in the **7OHQ** moiety. The relative energies of **K** in respect of **E** under various substitution in **7OHQ** are collected in Tables S3 (Supporting Information File 1) and the directions of change are illustrated in Figure 8a. It seems that substitution on positions 2 (with exception of a CH_3_ group) and 3 leads to destabilization of **K**, while placing a substituent in positions 5 (weakly) and 6 stabilizes it. The effect on position 4 is mixed – electron donors stabilize the **K** form, while acceptors rise its energy. All substituents in position 8 stabilize **K**, but the effect of the acceptors is more pronounced. The effect of the amino group is stronger comparing to NMe_2_ due to the steric hindrance in the latter. Since in **1** the azo group, an acceptor, is placed on position 8, it is also expended to bring additional inherent stabilization of the **1K** tautomer.

The effect of the substitution in the **7OHQ** part of **1** is illustrated in Figure 8b–d and the corresponding values are collected in Table S4, Supporting Information File 1. In **1** all three criteria for clean switching are not fulfilled and it is interesting to see how the substitution can change the situation. In respect of Δ*E*(**K**-**E**) (Figure 8b) the needed reduction is observed in position 2 for CH_3_ group (weakly), substantially for NR_2_ in position 4 and strongly for all substituents in position 6. The value of Δ*E*(**KE**-**E**) (Figure 8c) has to be larger than 2, but it cannot be achieved. Almost all functional groups on position 2 and all on position 4 lead to changes in the desired direction. The substitution on positions 5 and 6 leads to strong stabilization of **KE**, which is not needed. The cyano group on position 5 destabilizes **KE** strongly. The value of Δ*E*(**KK**-**K**) has also to be larger than 2 and, as seen from Figure 8d, it cannot be achieved with the used substituents. Most of the substituents destabilize **K** with exception of the electron donor ones on position 4 and all in position 6. Again, the cyano group on position 5 is acting in the desired direction, but the effect is weak. Considering the fact that some substituents act positively in some cases and negatively in others, it can be concluded substitution on position 4 by NR_2_ or CH_3_ and on position 5 by CN leads to desired changes in all three parameters. The effect on substitution in position 6 is contradictory in respect of Δ*E*(**KE**-**E**) and Δ*E*(**KK**-**K**).

Based on the previous knowledge about the relative stabilities of **E** and **KE** in **3**, it can be stated that electron-acceptor substituents in the phenyl ring shift the equilibrium towards **KE**, while electron donors do the same in direction of **E**. In this charge transfer model, the N=N group plays a role of acceptor, stabilized by donors in the phenyl ring, while N–NH is a donor, favored by acceptors in Ph [3,7,89]. Following this model, a suitable substitution in the Ph ring of **1** can stabilize or destabilized **KE** and **KK** in respect to **E** and **K**. This opens an additional channel for fulfilling the requirements for a clean switching. It should be noted, however, that the relative stabilization of **KE** and **KK** depends, in addition, on the strength of the intramolecular hydrogen bonding. The interaction C=O···H–N is much stronger, which leads to better stabilization of **KE**.

Some border cases of substitution in the phenyl ring of **1** are considered in Table 2. As expected, the substitution with electron-donor substituents destabilizes the **KE** and **KK** tautomers leading to changes of Δ*E*(**KE**-**E**) and Δ*E*(**KK**-**K**) in the desired direction. At the same time, they destabilize **K**, which is not desired. Para-substituted NO_2_ and CN compounds feature undesired stability of **KE** and **KK** (and hence, low values of Δ*E*(**KE**-**E**) and Δ*E*(**KK**-**K**)), but the increased number of CN substituents leads to a rise of Δ*E*(**KE**-**E**) and Δ*E*(**KK**-**K**) and, also, to the requested stabilization of **K**. Considering the data in Table 2, there is no compound that nears to the ground-state PES, requested for clean switching. Obviously, a cooperative effect between substitution in the Ph ring and the 7-OH quinoline part is needed.

**Table 2:** Relative stability of the tautomers of compound **1** in toluene as a function of the substituents in Ph ring.

Position	Subst.	Δ*E* [kcal/mol]				Δ*G*^0^ [kcal/mol]			
		**E**	**KE**	**KK**	**K**	**E**	**KE**	**KK**	**K**

	H	0.00	0.11	1.3	7.5	0.00	1.7	2.4	8.4
*p*	NO_2_	0.81	0.00	0.65	6.9	0.00	0.08	0.69	5.0
*p*	CN	0.52	0.00	0.77	6.9	0.00	0.67	1.4	6.1
*o*,*o*’	CN	0.00	1.6	0.54	2.7	0.00	0.82	0.13	2.1
*o*,*o*’,*p*	CN	1.1	1.4	0.00	1.9	0.17	0.91	0.00	1.5
*m*,*m*’	CN	0.00	0.15	0.81	5.7	0.00	1.4	1.9	5.4
*p*	NH_2_	0.00	1.6	3.2	8.5	0.00	2.0	3.8	8.8
*o*,*o*’,p	NH_2_	0.00	5.5	7.5	9.6	0.00	5.1	8.1	10
*p*	NMe_2_	0.00	1.8	3.5	8.7	0.00	2.4	4.7	9.8
*o*,*o*’,*p*	NMe_2_	0.00	2.7	4.5	9.1	0.00	2.5	4.8	9.5
*p*	N(*t*-Bu)_2_	0.00	0.35	1.6	7.7	0.00	0.86	2.2	8.0
julolidine		0.00	2.0	3.9	9.1	0.00	1.7	3.9	9.5
*o*,*o*’,*m*,*m*’,*p*	F	0.00	1.6	2.0	5.3	0.00	1.7	2.4	8.4

**Scheme 3:**
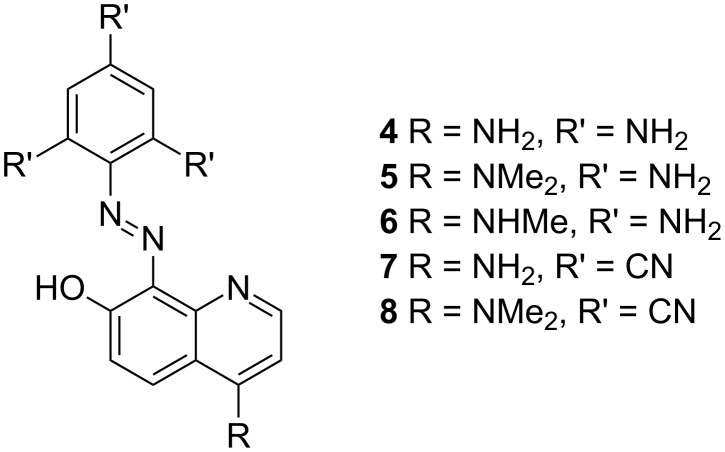
Perspective switching compounds, generated by the computational quantum chemistry calculations.

In Scheme 3 and Table 3 the most suitable examples for cooperative substitution are shown. It seems that the substitution with strong donor substituents in the phenyl ring and on position 4 in 7-OH quinoline leads to results that are near to the desired (compounds **4**–**6**). Very interesting is the result with strong electron-acceptor-substituted phenyl ring and donor substitution in **7OHQ** part (compounds **7** and **8**). Actually **7** and **8** fulfill most of the requirements for clean switching if the **K** tautomer is considered as off-state and the **E** one as on-state. The predicted spectra of the terminal **E** and **K** tautomers of compounds **4** and **7** are compared with those of compound **1** in Figure S13 in Supporting Information File 1. The increased charge-transfer character of compound **4** compared to **1** leads to a red shift in the absorption spectra of both tautomers. At the same time the long-wavelength maximum of **7K** is blue shifted in respect to **7E**, which in practical means would lead again to a red shift when switching from the more stable former to latter.

**Table 3:** Relative stability of the tautomers of compounds **4–7** in toluene.

compound	Δ*E* [kcal/mol]				Δ*G*^0^ [kcal/mol]			
	**E**	**KE**	**KK**	**K**	**E**	**KE**	**KK**	**K**

**4**	0.00	5.8	7.2	7.0	0.00	5.4	7.7	6.9
**5**	0.00	5.8	7.5	8.4	0.00	5.4	7.7	8.3
**6**	0.00	6.0	7.5	7.2	0.00	5.6	7.3	7.6
**7**	2.5	3.4	1.0	0.00	3.3	4.4	1.6	0.00
**8**	1.6	2.3	0.48	0.00	0.84	1.7	0.00	0.39

Of course, the results in this part should be considered with care. They originate from computational quantum chemistry calculations in toluene and their aim is not to suggest definite compounds, but to show directions of structural modifications that can solve one of the problems with the switching of compounds originating from **1** – the unsuitable relative stability of the ground-state tautomers. Changing the solvent can also change the results, because **KK** and, especially, **K** could be much more stabilized in polar solvents. The problem with avoiding the *cis*/*trans* isomerization, which reduces sharply the PT switching is also a problem pending solution.

## Conclusion

Two novel azo dyes originating from 7-hydroxyquinoline have been synthesized and studied by a variety of experimental and theoretical methods. It has been shown that 8-(phenyldiazenyl)quinolin-7-ol exists in solution as an equilibrium mixture of three different tautomers, whose proportion depends on the solvent environment. Upon irradiation reversible showing is detected in toluene, based on proton transfer and intramolecular switching. The reduced basicity in the azo nitrogen atoms in the case of 8-(4-hydroxy-1,2,3,5-tetrafluorophenyldiazenyl)quinolin-7-ol does not allow a ground-state tautomeric process to occur.

Bearing in mind the requirements for clean switching, where the tautomeric proton is transferred from the oxygen atom to the nitrogen atom of the quinolyl moiety (i.e. from **E** to **K**), a theoretical design was performed in order to see the most suitable substituents and their position. It seems that the substitution with strong donor substituents in the phenyl ring and on position 4 in 7-hydroxyquinoline leads to results that are near to the desired. Very interesting is the result with strong electron-acceptor-substituted phenyl ring and donor substitution in 7-OH quinoline, where the most of the requirements for clean switching are achieved if the **K** tautomer is considered as off-state and the **E** one as on-state.

## Experimental

### Synthesis and structure elucidation

#### Materials

The starting reagents and solvents were purchased from Sigma-Aldrich and Fluorochem and used as received. The solvents used for the experimental synthesis (HPLC grade) were purchased from Sigma-Aldrich. All other materials were commercial products of analytical grade and used as supplied. Analytical TLC (thin-layer chromatography) was performed on Merck silica gel 60 F254 precoated TLC plates (0.2 mm thick). The identity of all compounds was confirmed employing various spectroscopic techniques including NMR and HRMS–ESI high-resolution mass spectrometry. The ^1^H and ^13^C NMR spectra were recorded in CDCl_3_ or DMSO-*d*_6_ at 25 °C on a Bruker Ultrashield Plus 500 spectrometer using 5 mm tubes. The corresponding operating frequencies were 500.13 MHz and 125.77 MHz, respectively. The chemical shifts are quoted in parts per million (ppm) with an accuracy of 0.01 ppm. The coupling constants (*J*) are described in Hz and determined with an accuracy of 0.1 Hz. To describe the spin multiplicity in the ^1^H NMR spectra, the following abbreviations were used: s = singlet, br s = broad singlet, d = doublet, t = triplet, dt = double triplet, dd = doublet of doublets, m = multiplet. The HRMS spectra were recorded on a Dionex Acclaim RSLC 120 C18 2.2 mm 120 Å 2.1 × 50 mm column maintained at 40 °C. The measurements were done on a Bruker MicrOTOF-QII-system coupled with an ESI source with a nebulizer: 1.2 bar, dry gas 8.0 L min^−1^, dry temperature 200 °C, capillary 4500 V and plate offset 500 V.

#### Synthesis of 8-(phenyldiazenyl)quinolin-7-ol (**1**)

A 50 mL round-bottomed flask equipped with a mini magnet was charged with aniline (0.33 g, 3.5 mmol) and 1 N aqueous HCl (12 mL), and stirred at 0 °C for 30 min. A cold solution of sodium nitrite (0.38 g, 5.5 mmol, dissolved in 4.0 mL H_2_O) was added dropwise during 10 min and the resulting mixture was stirred at 0 °C for 2 h. The mixture was diluted by adding pre-cooled methanol (4.0 mL). In a separate round-bottomed flask, 7-hydroxyquinoline (0.29 g, 2.0 mmol) and potassium hydroxide (0.163 g, 3.0 mmol) were dissolved in methanol (6.0 mL) and cooled to 0 °C. This solution was treated under constant stirring by dropwise addition of the cold diazonium solution during 20 min. The resulting red-orange mixture was stirred at 0 °C for 2 h, and at rt for 15 h. The mixture was neutralized with 1 N aqueous sodium hydroxide, filtered, washed with cold water (5 mL) and dried under vacuum. The dark orange crude material was purified by flash column chromatography on silica gel, using dichloromethane/ethyl acetate 6:1 as eluent to obtain the pure product as bright orange solid (0.31 g, 62% yield). ^1^H NMR (500 MHz, CDCl_3_) δ 8.93 (dd, *J* = 1.2 Hz, 4.6 Hz, 1H), 7.95 (d, *J* = 8.0 Hz, 1H), 7.87 (d, *J* = 8.0 Hz, 2H), 7.70 (d, *J* = 9.5 Hz, 1H), 7.49 (t, *J* = 8.0 Hz, 2H), 7.73–7.33 (m, 2H), 7.02 (d, *J* = 9.5 Hz, 1H); ^13^C NMR (125 MHz, CDCl_3_) δ 171.3, 151.3, 148.5, 145.0, 137.7, 136.5, 131.0, 129.7, 128.8, 125.8, 123.2, 120.7, 120.0; HRMS–ESI (*m*/*z*): [M + H]^+^ calcd for C_15_H_12_N_3_O^+^, 250.09749; found, 250.09739.

#### Synthesis of 8-(4-hydroxy-1,2,3,5-tetrafluorophenyldiazenyl)quinolin-7-ol (**2**)

Diazotization of pentafluoroaniline has been recommended to be carried out in nonaqueous media or concentrated mineral acid, otherwise the coupling product always contains a hydroxy group on the perfluorophenyl ring at the position para to the azo group. It appears that when solutions of the diazonium salts are made alkaline the para-fluorine atom is so readily replaced by a hydroxy group, and that the 4-hydroxy-2,3,5,6-tetrafluorobenzene cation is the entity formed first [90–91]. A 50 mL round-bottomed flask equipped with a mini magnet was charged with pentafluoroaniline (0.64 g, 3.5 mmol) and 1 N aqueous HCl solution (12.0 mL) and methanol (7.0 mL). The mixture was stirred at rt for 2 h, then cooled in an ice bath to 0 °C, and treated slowly with a cold solution of sodium nitrite (0.38 g, 5.5 mmol) in distilled water (3.0 mL). The mixture was left stirring at 0 °C for 2 h. In a separate 50 mL flask, 7-hydroxyquinoline (0.29 g, 2.0 mmol) dissolved in methanol (4.0 mL) was treated with potassium hydroxide (0.16 g, 3.0 mmol). The solution was stirred at 0 °C for 20 min, then the cooled diazonium salt solution was added slowly to the cold solution of 7-hydroxyquinoline during 30 min. Upon addition, a red-orange color formed. The mixture was left stirring at 0 °C for 2 h, then at rt overnight. The mixture was filtered, the filtrate was neutralized with 1 N aqueous potassium hydroxide solution, diluted with dichloromethane (75 mL) and stirred overnight. The orange solid formed, was washed with water several times and dried. The crude material was purified by column chromatography on silica gel, eluting with dichloromethane. Evaporation of the solvent afforded the pure target compound as orange solid (0.26 g, 38% yield). ^1^H NMR (500 MHz, DMSO-*d*_6_) δ 14.7 (br s, 1H), 8.90 (dd, *J* = 1.7, *J* = 4.3, 1H), 8.28 (dd, *J* = 1.7, *J* = 8.2, 1H), 7.86 (d, *J* = 9.2 Hz, 1H), 7.44 (dd, *J* = 4.2 Hz, *J* = 4.2 Hz, 1H), 7.25 (d, *J* = 9.2 Hz, 1H); ^13^C NMR (125 MHz, DMSO-*d*_6_) δ 156.5, 153.0, 151.0, 145.2, 144.0 (d, *J* = 240 Hz), 141.2 (d, *J* = 240 Hz), 136.5, 131.2, 131.1, 123.0, 120.9, 120.0, 110.5; ^19^F NMR (470 MHz, DMSO-*d*_6_) δ 154.5 (d, *J* = 17.7 Hz), −169.2 (d, *J* = 17.7 Hz); HRMS–MALDI–TOF [*m*/*z*]: [M + H]^+^ calcd for C_15_H_8_F_4_N_3_O_2_^+^, 338.05471; found, 338.05477.

Spectral investigations: The NMR spectra were recorded on a Bruker Avance II+ spectrometer operating with frequency 500 MHz for ^1^H NMR and 125 MHz for ^13^C NMR in CD_2_Cl_2_ and 600 MHz for ^1^H NMR and 151 MHz for ^13^C NMR in CDCl_3_ and CD_3_CN. ATR-FTIR spectra of the compounds were recorded on a Bruker Tensor 27 FTIR spectrophotometer in the range of 4400–600 cm^−1^ with a resolution of 2 cm^−1^ at room temperature. The external reflection diamond crystal was used and the samples were scanned 128 times. The UV–vis spectra were measured on a Jasco V-570 UV–vis–NIR spectrophotometer using spectral grade solvents in the concentration range ≈10^−5^ mol/L. The steady-state fluorescence spectra were recorded with a FluoroLog 3-22 (HORIBA) spectrofluorometer in the range 200–800 nm with a resolution of 0.5 nm and double-grating monochromators using as excitation wavelength a value near the absorption maxima of the compounds with concentrations of ≈10^−6^ mol L^−1^.

The irradiation experiments were performed with an experimental setup designed and constructed by us [92]. The configuration allows to use two light sources whose beams path orthogonally through a cuvette. The absorbance spectra were measured using a fiber-optic micro-spectrometer QE 65000 (Ocean Optics, Inc., Dunedin, FL, USA) with approximately 0.7 nm spectral resolution in the range 250–1000 nm. The spectra were recorded with the specialized software SpectraSuite (Ocean Optics, Inc., Dunedin, USA).

Computational quantum chemistry calculations: Quantum-chemical calculations in the ground state were performed using the Gaussian 16 C.01 program suite [93]. All structures (in both ground and excited state) were optimized without restrictions, using tight optimization criteria and an ultrafine grid in the computation of two-electron integrals and their derivatives. The true minima were verified by performing frequency calculations in the corresponding environment. The implicit solvation was described using the polarizable continuum model [94] (the integral equation formalism variant, IEFPCM, as implemented in Gaussian 16). The transition states were estimated using the STQN method [95] and again verified by performing frequency calculations in the corresponding environment.

The M06-2X [96–97] functional with the TZVP [98] basis set was used for the structure optimizations in the ground state. The use of M06-2X provides very good predictability in the ground state [51,53,99–103] of the tautomeric composition in tautomeric compounds and proton cranes as well as the *E*/*Z* isomerization ratio in rotary switches. In compound **1** the obtained values for the relative energies are validated by using Møller-Plesset MP2-4 [104–107] and CCSD(T) [108–109] single point energies (M06-2X/TZVP geometry) in the corresponding solvent environment.

The TD-DFT method [110–111] was used for singlet excited-state optimizations. CAM-B3LYP [112] with the TZVP basis set was used for the optimizations. The selection of CAM-B3LYP is based on its better performance (in comparison with a variety of density functionals, including M06-2X) in describing electronic excitation energies, excited-state geometries, dipole moments and oscillator strengths in a variety of systems [113–116], including ESIPT ones [117], as well as on our own previous experience in describing the overall shape of the excited-state PES [51–52,92].

Bearing in mind that M06-2X systematically underestimates the absorption band positions [118], the UV–vis spectral data were predicted by the B3LYP [119] functional (TZVP basis set) using the M06-2X optimized ground-state geometries.

The NMR chemical shieldings of selected tautomeric forms of the studied compounds were calculated using the GIAO approximation [120]. The calculated absolute shieldings were transformed to chemical shifts using the reference compound tetramethylsilane (Si(CH_3_)_4_), for hydrogen and carbon atoms: δ = δ_calc_(ref) − δ_calc_. Both δ_calc_(ref) and δ_calc_ were evaluated at the same computational level (M06-2X/TZVP).

## Supporting Information

File 1 Additional figures and tables.

## Data Availability

Data generated and analyzed during this study is available from the corresponding author upon reasonable request.
