# Supplementary material for: Tautomerism and switching in 7-hydroxy-8-(azophenyl)quinoline and similar compounds
Source: Beilstein J Org Chem. 2025 Jul 10;21:1404–21. doi: 10.3762/bjoc.21.105 (PMC12256787; doi:10.3762/bjoc.21.105)
Supplement: File 1 — Additional figures and tables. [file Beilstein_J_Org_Chem-21-1404-s001.pdf]

BEILSTEIN JOURNAL OF ORGANIC CHEMISTRY

Supporting Information

for

Tautomerism and switching in 7-hydroxy-8-(azophenyl)quinoline and similar compounds

Lidia Zaharieva, Vera Deneva, Fadhil S. Kamounah, Nikolay Vassilev, Ivan Angelov, Michael Pittelkow and Liudmil Antonov

Beilstein J. Org. Chem. **2025,** *21,* 1404–1421. doi:10.3762/bjoc.21.105

Additional figures and tables

License and Terms: This is a supporting information file under the terms of the Creative Commons Attribution License (https://creativecommons.org/licenses/by/4.0). Please note that the reuse, redistribution and reproduction in particular requires that the author(s) and source are credited and that individual graphics may be subject to special legal provisions.
The license is subject to the *Beilstein Journal of Organic Chemistry* terms and conditions: (https://www.beilstein-journals.org/bjoc/terms)

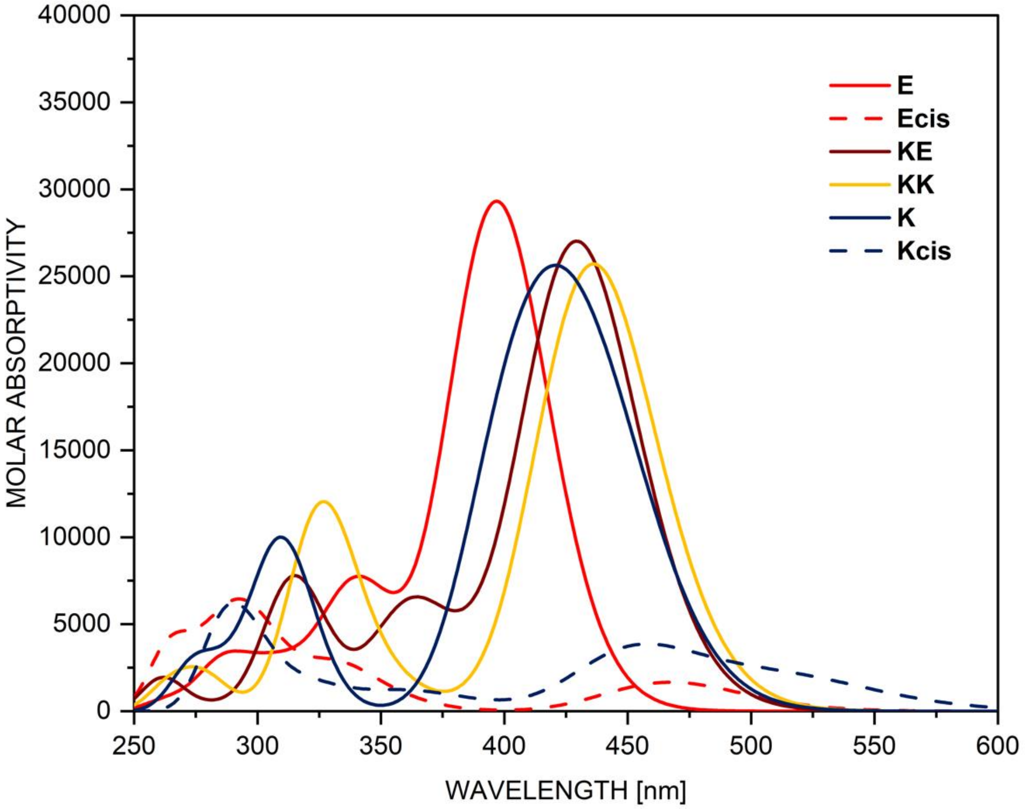

Figure S1. Simulated absorption spectra of the tautomers and isomers of **1** in acetonitrile.

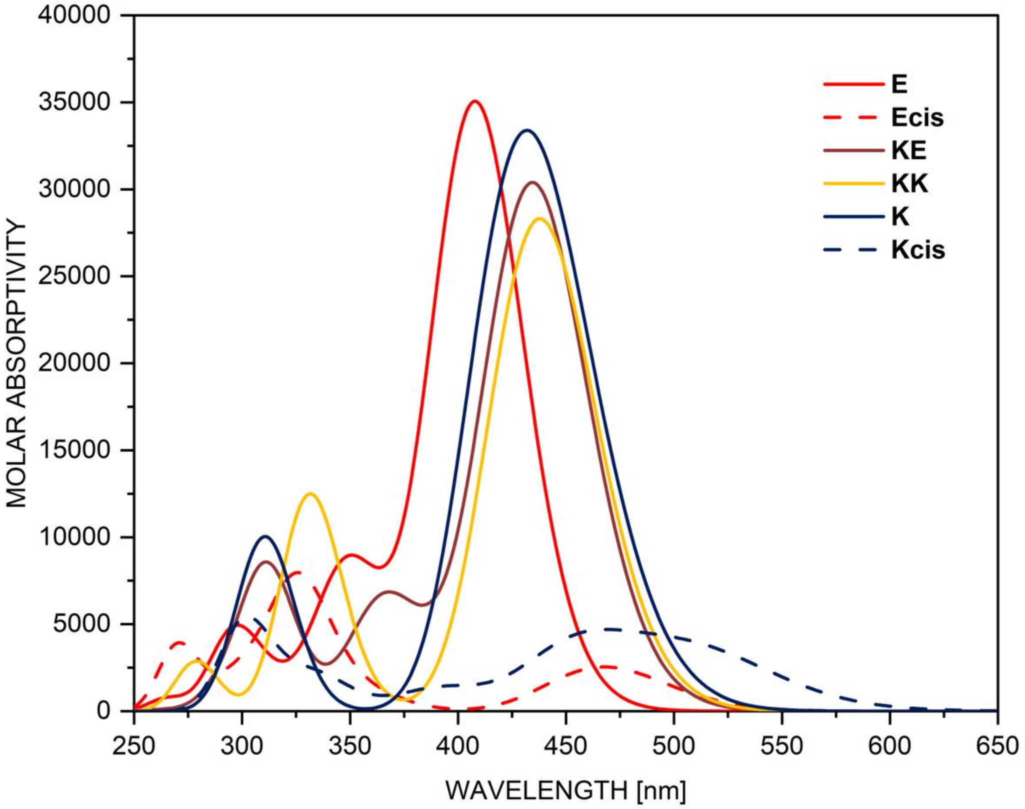

Figure S2. Simulated absorption spectra of the tautomers and isomers of **2** in toluene.

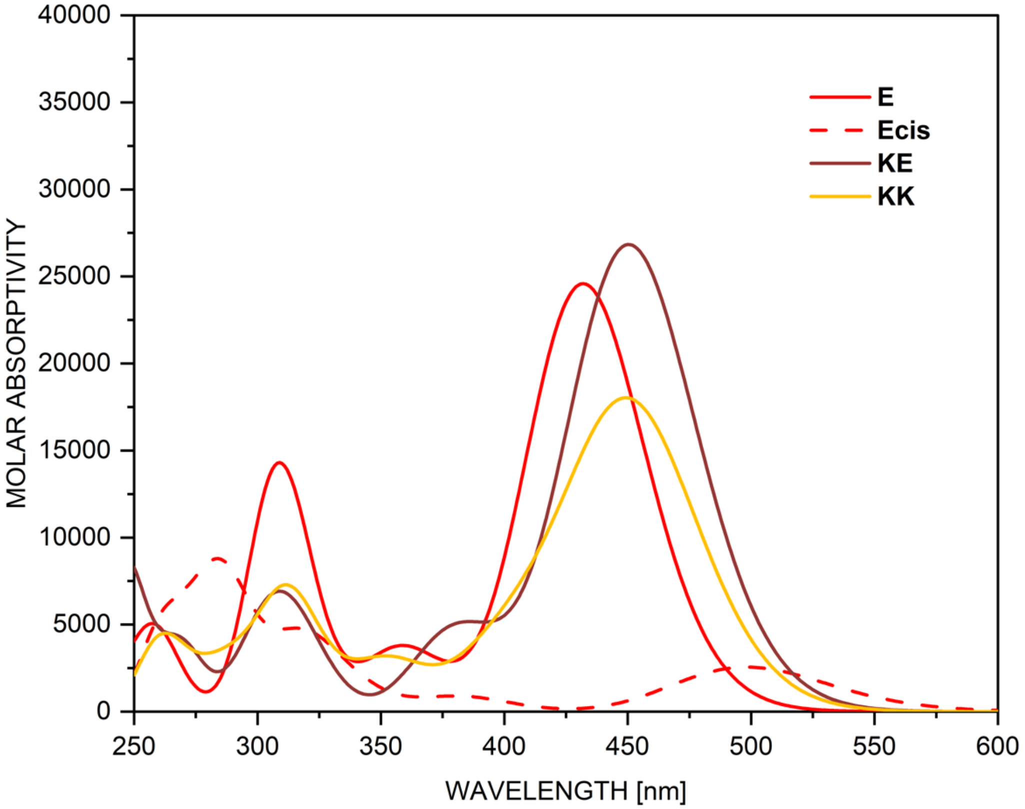

Figure S3. Simulated absorption spectra of the tautomers of **3** in toluene.

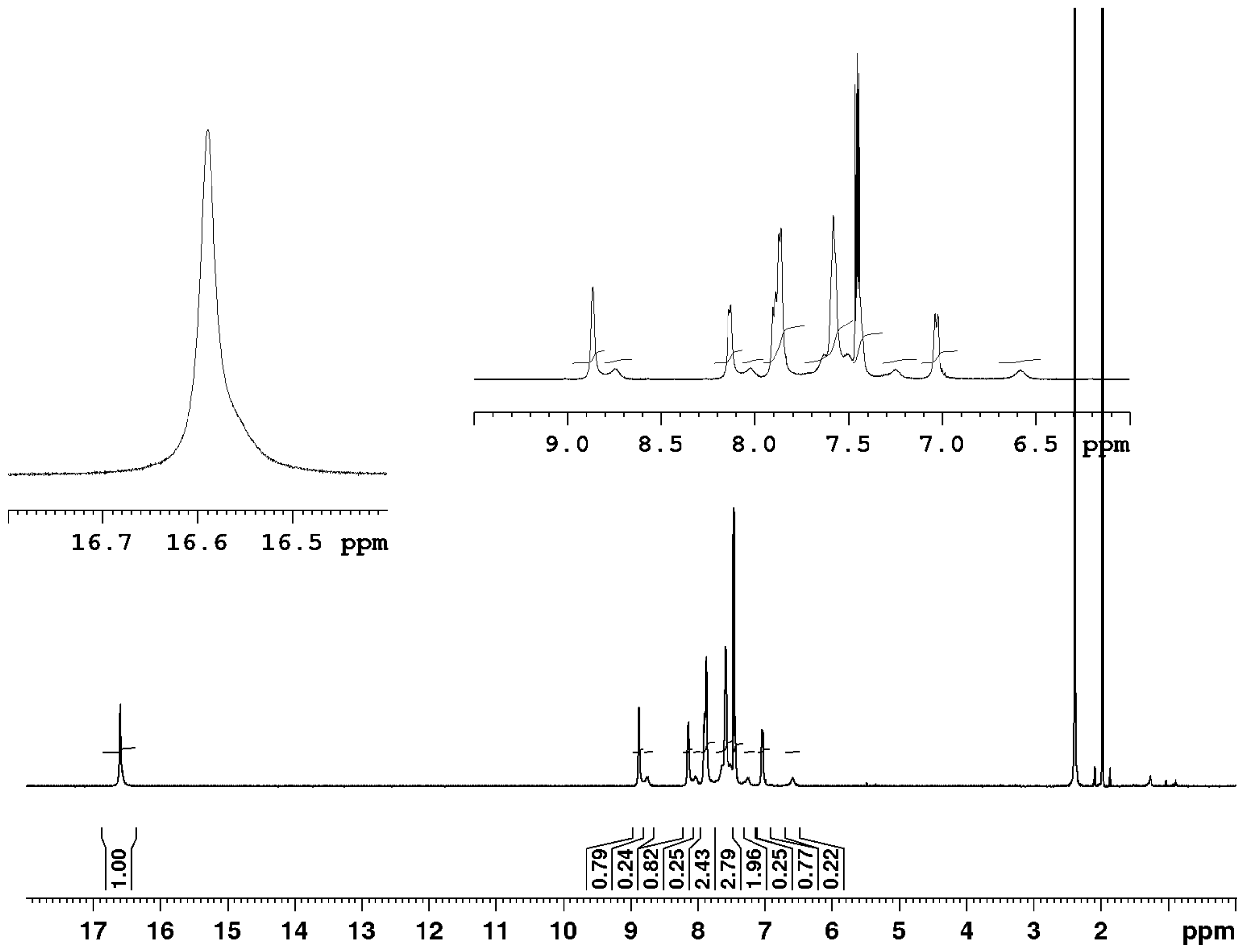

Figure S4. ^{1}H NMR spectrum of **1** in acetonitrile-d_3 at 243 K.

^{1}H NMR (CD_3CN, 243 K): Signals of major tautomeric form: δ = 7.03 (bd, *J*=8.8 Hz, 1H, H-6), 7.44 (s, 1H, p-Ph), 7.46 (dd, J=4.5, 7.9 Hz, 1H, H-3), 7.58 (bs, 2H, m-Ph), 7.87 (bd, J=6.9 Hz, 2H, o-Ph), 7.90 (bd, J=9.5 Hz, 1H, H-5), 8.14 (d, J=6.7 Hz, 1H, H-4), 8.87 (s, 1H, H-2), 16.59 (bs, 1H, OH); Resolved signals of minor tautomeric form: 6.58 (bs, 1H, H-6), 7.25 (bs, 1H, p-Ph), 7.50 (bs, 1H, m-Ph), 7.63 (bs, 1H, o-Ph), 8.02 (bs, 1H, H-4), 8.75 (bs, 1H, H-2).

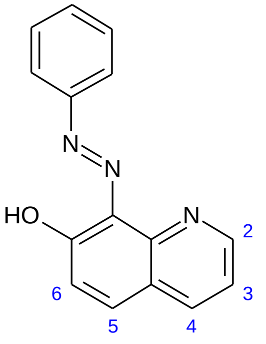

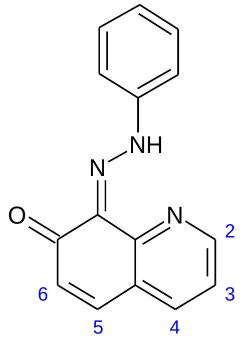

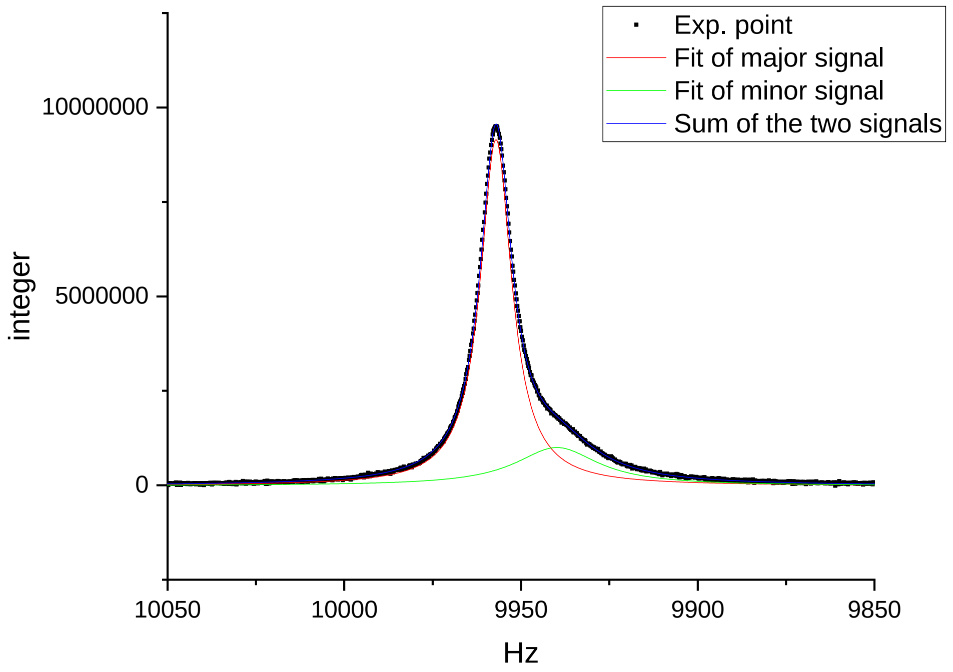

Figure S5. Deconvolution of two overlapped signals at 16.59 ppm in ^{1}H NMR spectrum of **1** in acetonitrile-d_3 at 243 K. The integrals of fitted signals are in relation: 78:22.

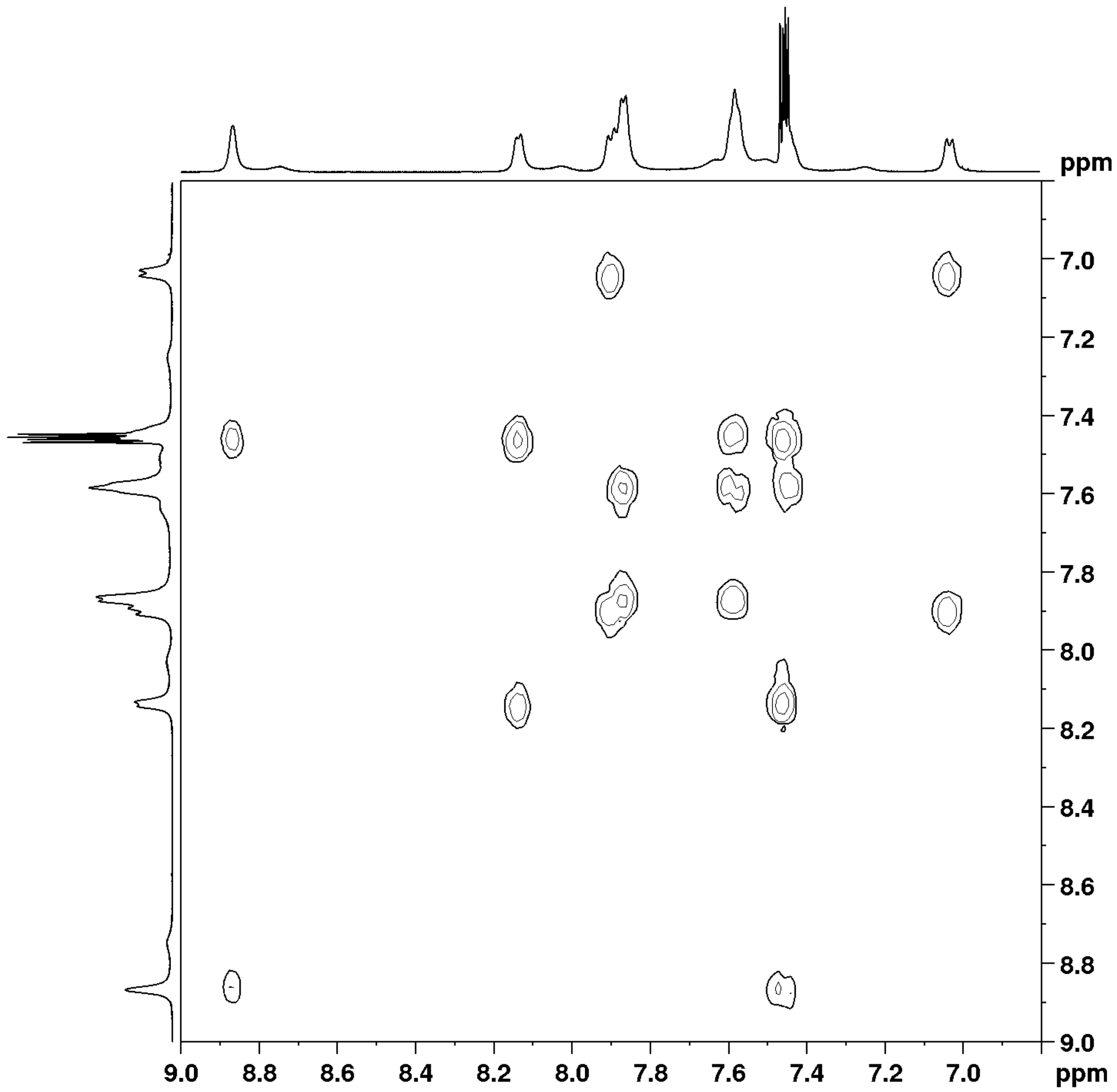

Figure S6. COSY spectrum of **1** in acetonitrile-d_3 at temperature 243 K.

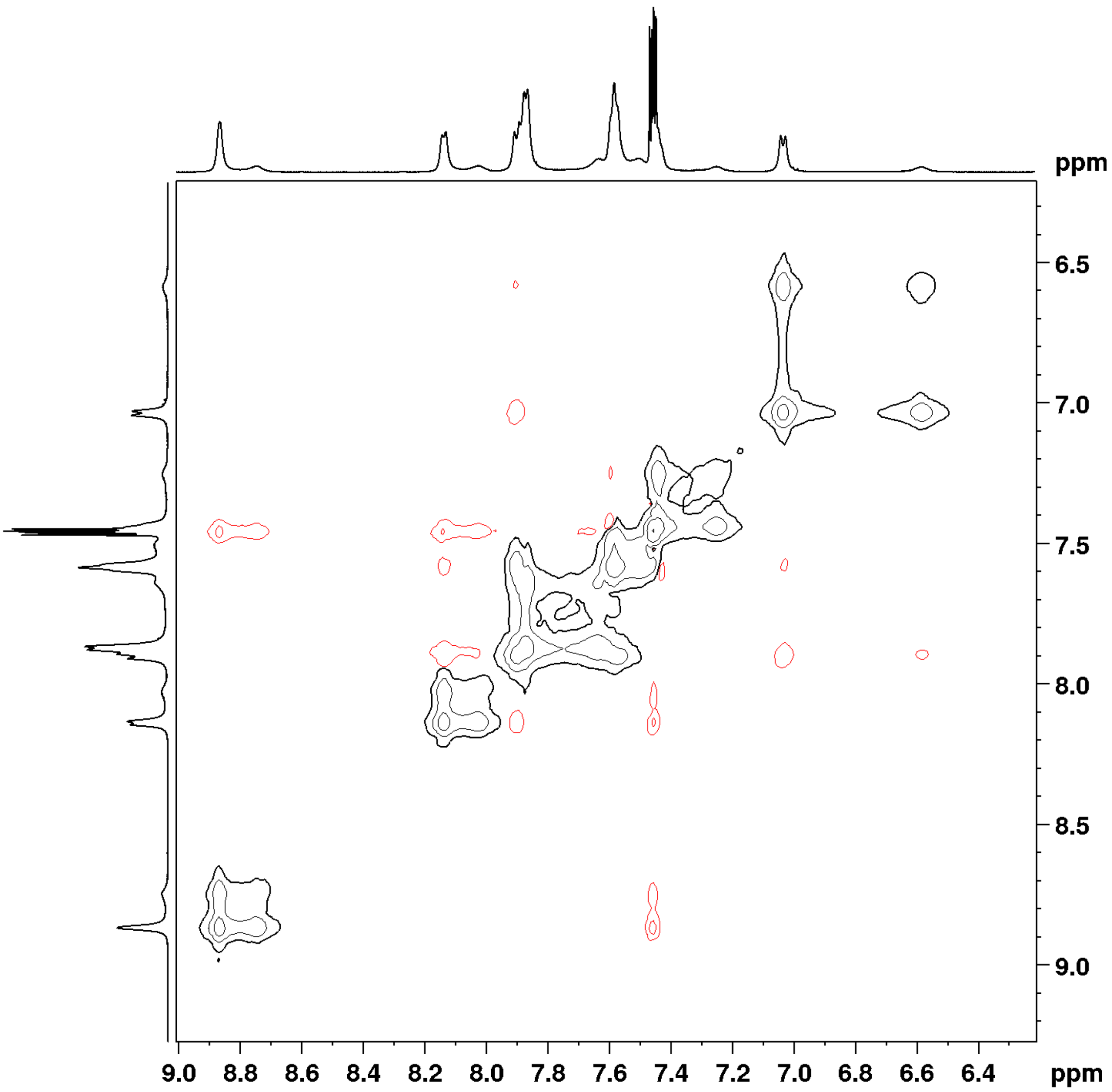

Figure S7. NOESY spectrum of **1** in acetonitrile-d_3 at temperature 243 K.

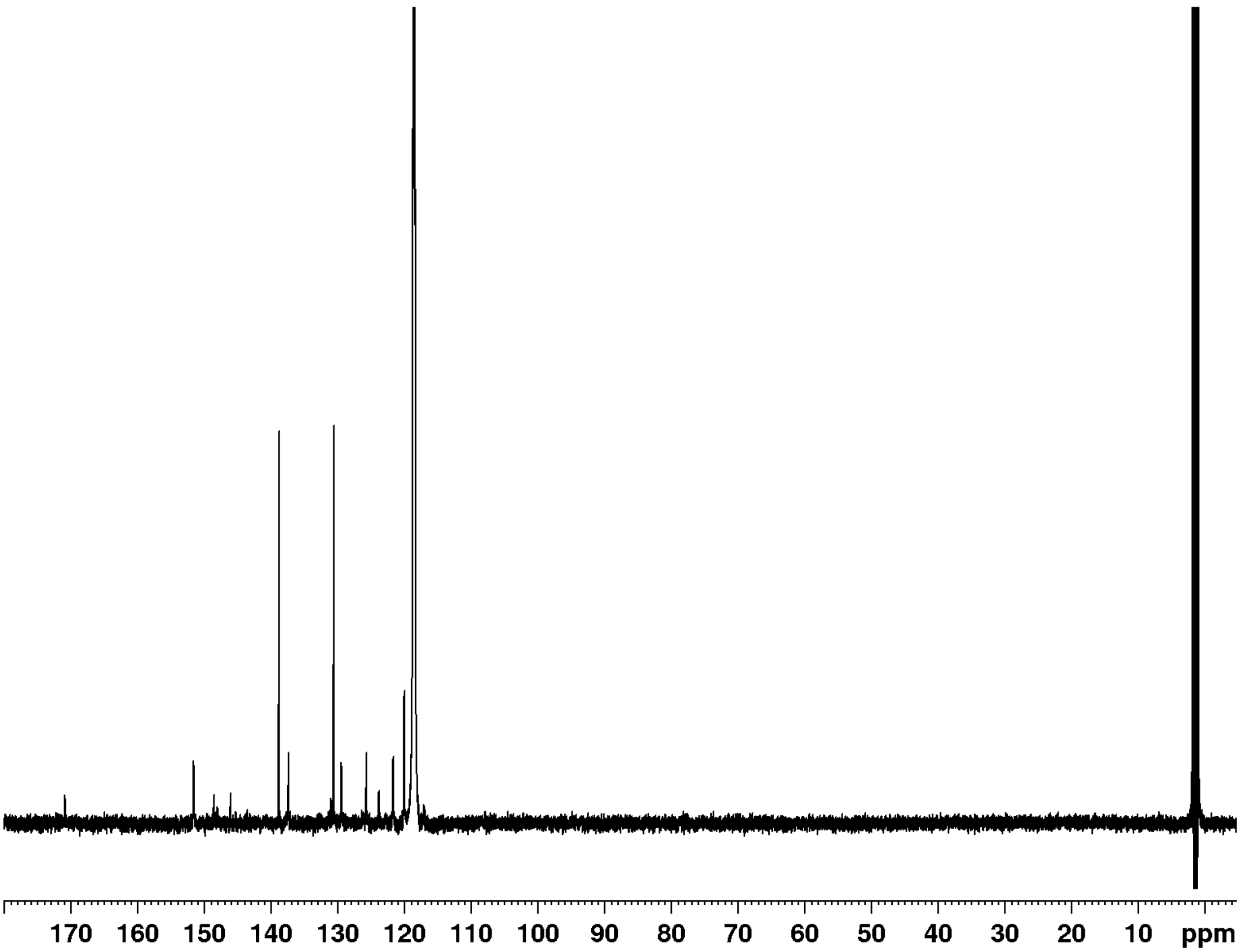

Figure S8. ^{13}C NMR spectrum of **1** in acetonitrile-d_3 at temperature 243 K.

^{13}C NMR (CD_3CN, 243 K): Signals of major component: δ = 120.03 (o-Ph), 121.69 (C-3), 125.71 (C-6), 129.46 (p-Ph), 130.61 (m-Ph), 137.37 (C-4), 138.82 (C-5), 151.62 (C-2).

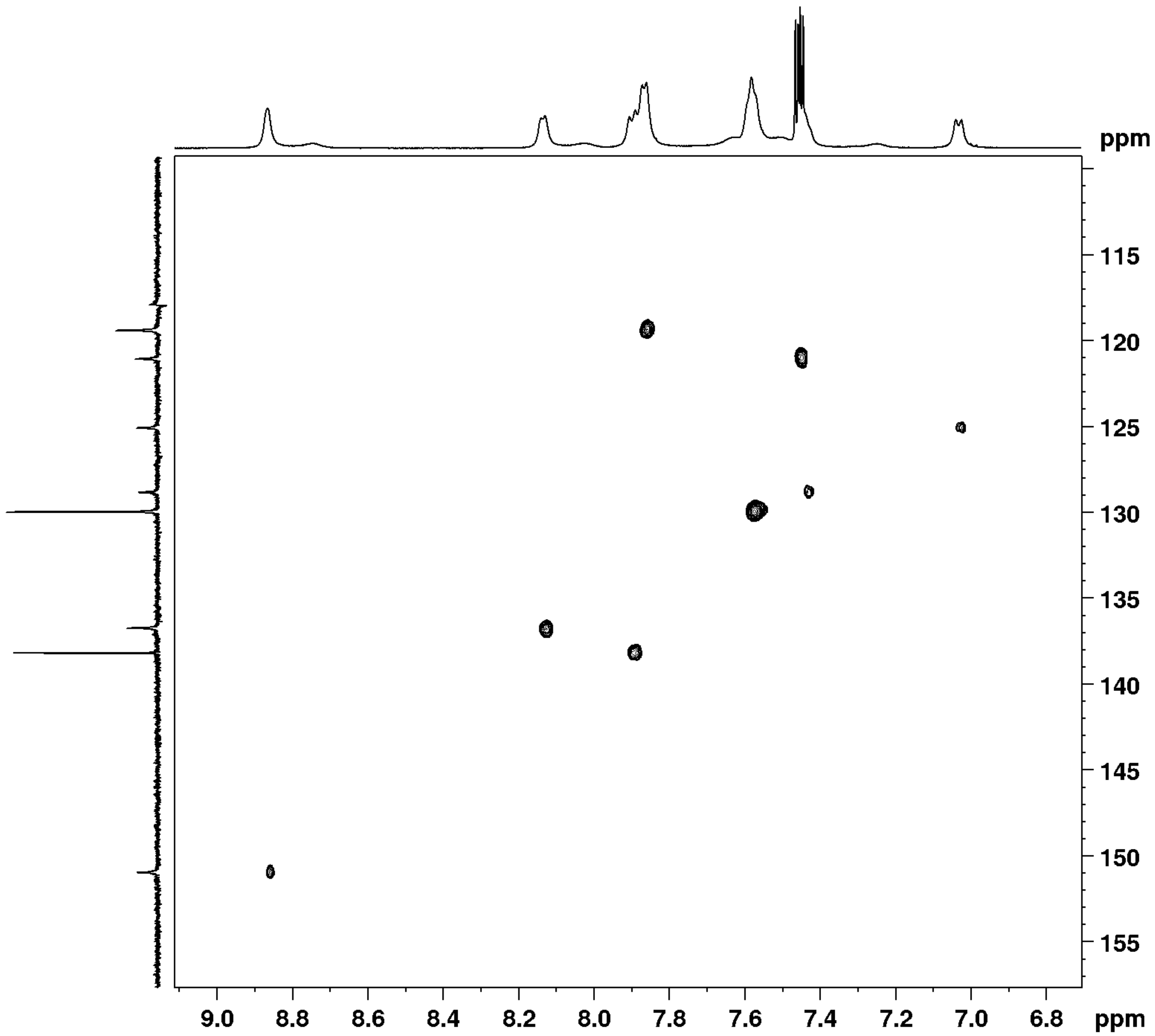

Figure S9. HSQC spectrum of **1** in acetonitrile-d_3 at temperature 243 K.

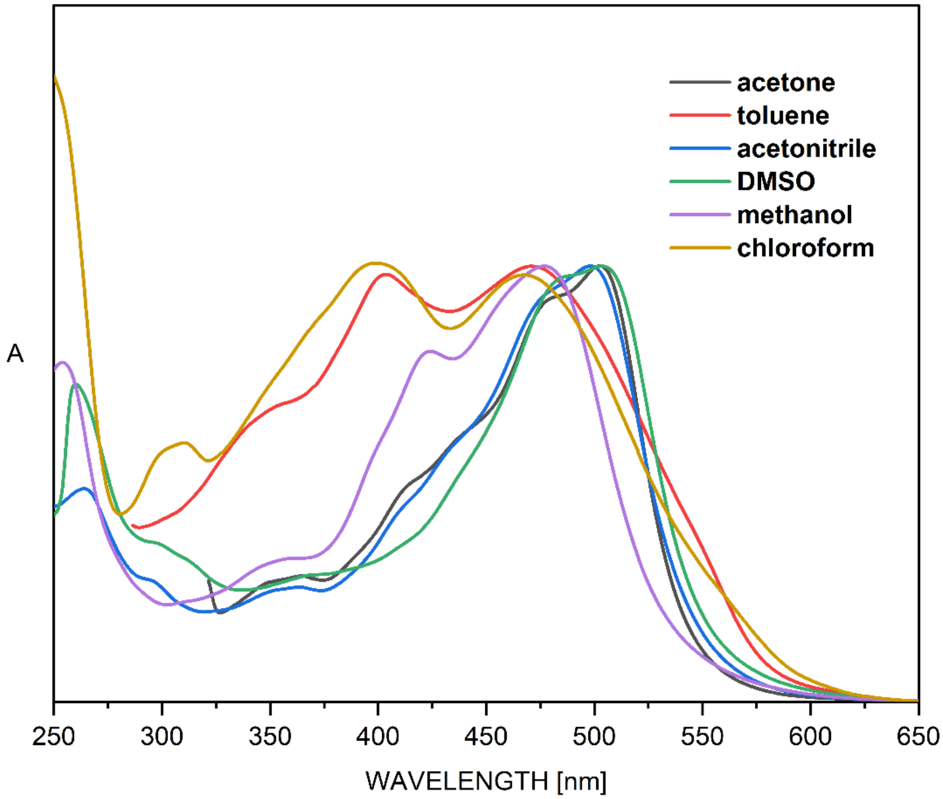

Figure S10. Normalized absorption spectra of **2**.

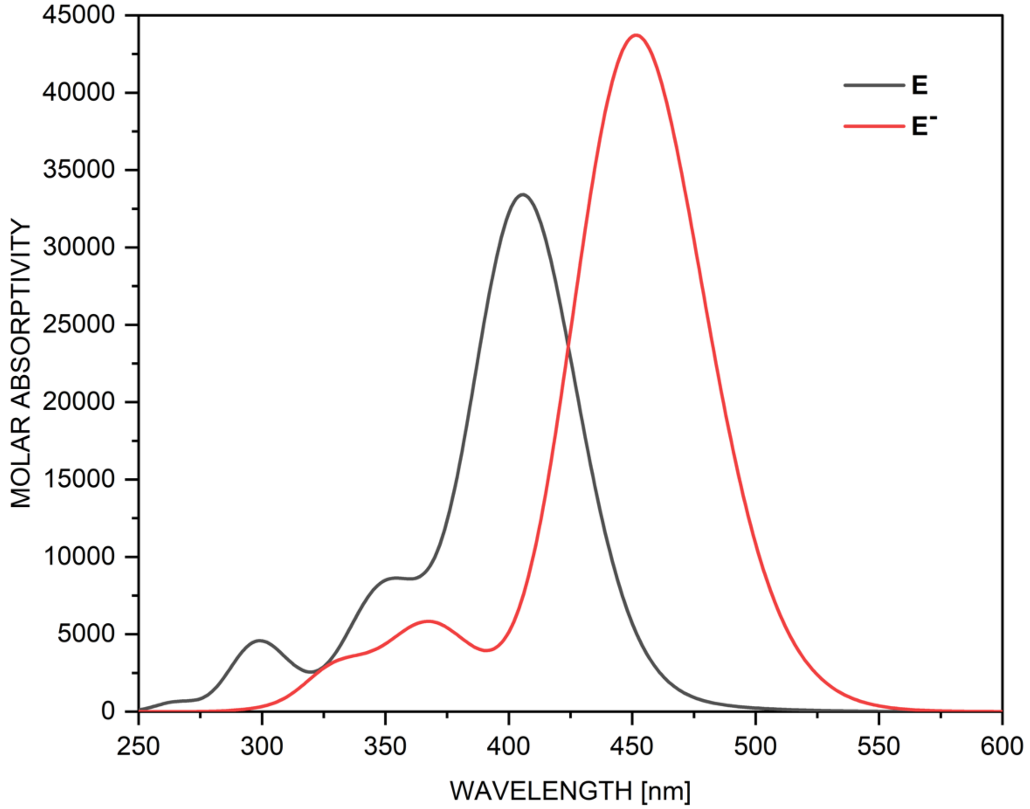

Figure S11. Comparison between the simulated spectra of **2E** and **2E**$^{-}$ in acetonitrile.

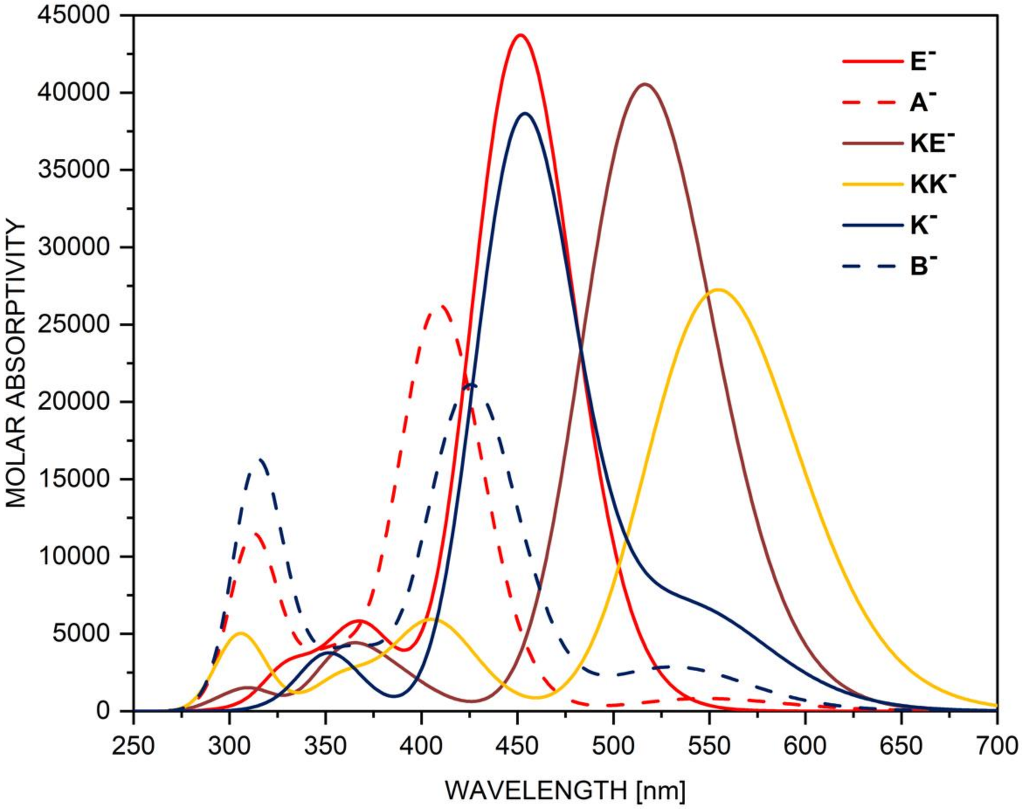

Figure S12. Simulated spectra of **2**⁻ in acetonitrile. The abbreviations are given in Table S2.

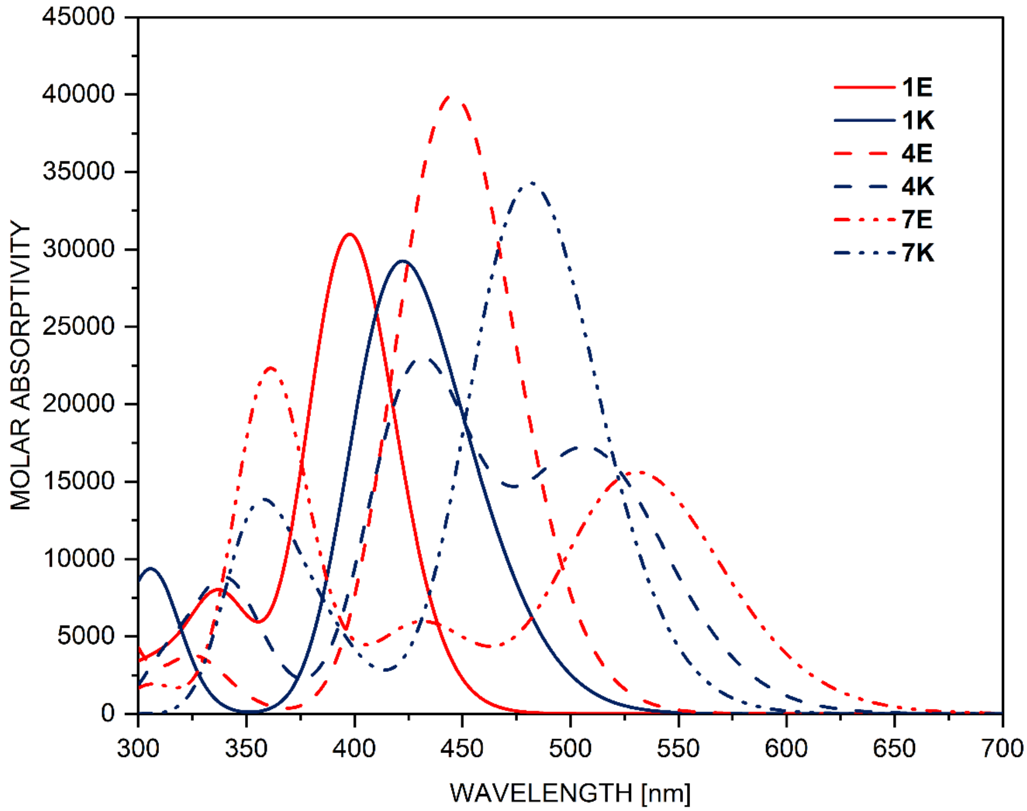

Figure S13. Simulated spectra of the end tautomeric forms of **1**, **4** and **7**⁻ in toluene.

Table S1. Comparison of the relative energies (ΔE) of the tautomers and of the transition states (in kcal/mol units) of **1** in the ground state in toluene and in acetonitrile (in brackets), obtained by a variety of methods.

Structure	M06-2X/ TZVP	MP2/ TZVP*	MP3/ TZVP*	full MP4/ TZVP*	CCSD/ TZVP*	CCSD(T)/ TZVP*
**E_{cis}**	17 (16)	10 (9.2)	12 (11)	10 (9.1)	11 (10)	11 (9.7)
**TS(E-E_{cis})**	49 (49)	52 (52)	53 (53)	51 (51)	52 (52)	52 (51)
**E**	0.0 (0.28)	0.0 (0.0)	0.0 (0.0)	0.0 (0.0)	0.0 (0.0)	0.0 (0.0)
**TS(E-KE)**	3.9 (3.9)	3.8 (3.6)	7.5 (7.3)	3.4 (3.3)	6.4 (6.2)	4.8 (4.6)
**KE**	0.11 (0.0)	2.15 (1.8)	2.8 (2.4)	0.37 (0.07)	1.4 (1.2)	1.3 (1.0)
**TS(KE-KK)**	41 (35)	41 (36)	41 (35)	38 (33)	39 (33)	39 (33)
**KK**	1.3 (0.16)	3.1 (1.8)	3.2 (2.0)	1.3 (0.11)	1.9 (0.70)	2.2 (0.95)
**TS(K-KK)**	9.8 (7.1)	11 (7.8)	14 (11)	9.6 (7.0)	13 (10)	11 (8.6)
**K**	7.5 (3.8)	9.5 (5.9)	9.9 (6.1)	7.6 (4.2)	8.7 (5.0)	8.3 (4.9)
**TS(K-K_{cis})**	54 (-)	59 (-)	61 (-)	56 (-)	59 (-)	57 (-)
**K_{cis}**	27 (22)	23 (18)	25 (20)	20 (16)	23 (18)	22 (17)

* single point calculations using M06-2X/TZVP geometries with TZVP basis set in the corresponding solvent environment.

Table S2. Relative stability (M06-2X/TZVP) and spectral characteristics of the ground-state tautomers and isomers of the deprotonated **2** in acetonitrile.

Structure	ΔE [kcal/mol]	ΔG^0 [kcal/mol]
**E⁻** 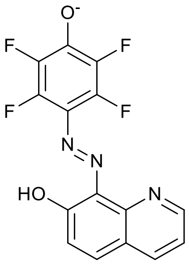	0.0	0.0
**KE⁻** 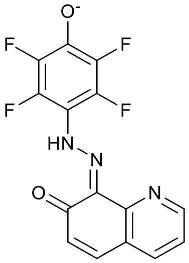	4.7	4.2
**KK⁻** 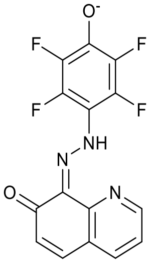	5.5	5.8
**K⁻** 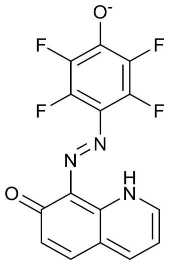	5.4	4.9

**A**$^-$ 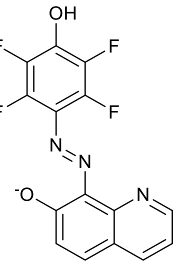	15	14
**B**$^-$ 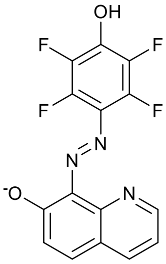	16	16

Table S3. Relative stabilization of **E** and **K** tautomers of **7OHQ** in toluene as a function of the substituents.

Subst.	ΔE [kcal/mol]		ΔG^0 [kcal/mol]	
	**E**	**K**	**E**	**K**
H	0.0	11	0.0	12
2-CN	0.0	16	0.0	17
3-CN	0.0	12	0.0	13
4-CN	0.0	13	0.0	13
5-CN	0.0	11	0.0	11
6-CN	0.0	9.3	0.0	9.5
8-CN	0.0	6.8	0.0	6.8
2-NH_2 *	0.0	15 (9.4)	0.0	15 (9.6)
3-NH_2	0.0	15	0.0	16
4-NH_2 *	0.0	8.5 (10)	0.0	7.6 (10)
5-NH_2 *	0.0	11 (25)	0.0	12 (25)
6-NH_2	0.0	5.9	0.0	6.4
8-NH_2	0.0	11	0.0	11
2-NMe_2	0.0	13	0.0	13
3-NMe_2	0.0	14	0.0	14
4-NMe_2	0.0	9.9	0.0	10
5-NMe_2	0.0	11	0.0	11
6-NMe_2	0.0	7.8	0.0	8.4
8-NMe_2	0.0	11	0.0	10
2-F	0.0	19	0.0	20
3-F	0.0	15	0.0	16
4-F	0.0	12	0.0	12
5-F	0.0	11	0.0	11
6-F	0.0	8.8	0.0	10
8-F	0.0	11	0.0	13
2-Cl	0.0	17	0.0	18
3-Cl	0.0	14	0.0	15
4-Cl	0.0	12	0.0	12
5-Cl	0.0	11	0.0	9.9
6-Cl	0.0	8.9	0.0	9.3
8-Cl	0.0	9.5	0.0	9.7
2-CF_3	0.0	17	0.0	17
3-CF_3	0.0	12	0.0	13
4-CF_3	0.0	14	0.0	14
5-CF_3	0.0	11	0.0	11

6-CF_3	0.0	9.4	0.0	9.8
8-CF_3	0.0	8.2	0.0	7.7
2-CH_3	0.0	11	0.0	11
3-CH_3	0.0	12	0.0	12
4-CH_3	0.0	11	0.0	11
5-CH_3	0.0	11	0.0	12
6-CH_3	0.0	10	0.0	9.8
8-CH_3	0.0	11	0.0	11

* NH_2 group on positions 2, 4 and 5 opens possibility for additional tautomer, **KN** (see the scheme below in the case of 4-substituted compound as an example). The relative energies are given in brackets below the values for **K** in the table.

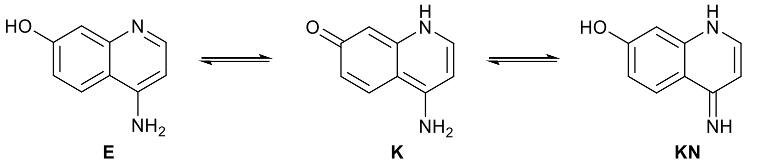

Table S4. Relative stabilization of the tautomers of **1** in toluene as a function of the substituents in 7-hydroxyquinoline part.

Subst.	ΔE [kcal/mol]				ΔG^0 [kcal/mol]			
	**E**	**KE**	**KK**	**K**	**E**	**KE**	**KK**	**K**
H	0.0	0.11	1.3	7.6	0.0	1.7	2.4	8.4
2-CN	0.0	0.04	2.2	13	0.43	0.0	3.3	14
3-CN	0.0	0.19	2.6	10	0.0	0.81	3.0	11
4-CN	0.0	0.36	2.7	10	0.0	1.8	3.8	11
5-CN	0.0	0.89	2.0	7.7	0.0	1.8	3.4	7.9
6-CN	0.85	0.0	1.4	7.2	0.0	0.0	1.2	6.8
2-NH_2 *	0.0<br>(6.0)	0.68<br>(9.8)	3.0<br>(23)	11<br>(7.8)	0.0<br>(6.4)	0.59<br>(10)	3.5<br>(23)	10<br>(8.4)
3-NH_2	0.0	0.04	0.72	9.4	0.0	0.24	1.4	9.8
4-NH_2 *	0.0<br>(5.3)	0.44<br>(8.8)	0.99<br>(20)	4.7<br>(6.8)	0.0<br>(5.6)	1.1<br>(9.7)	1.1<br>(20)	3.2<br>(7.1)
5-NH_2 *	1.5<br>(17)	0.0<br>(21)	2.3<br>(5.3)	10<br>(15)	0.99<br>(17)	0.0<br>(22)	2.2<br>(5.3)	10<br>(15)
6-NH_2	2.4	0.67	0.0	4.4	1.9	0.45	0.0	4.2
2-NMe_2	0.0	0.51	1.7	8.1	0.0	1.4	2.9	9.2
3-NMe_2	0.14	0.0	0.42	8.9	0.0	0.69	0.53	9.0
4-NMe_2	0.0	0.40	1.3	5.8	0.0	0.65	1.2	6.0
5-NMe_2	1.4	0.0	1.7	9.1	0.66	0.0	1.9	8.2
6-NMe_2	1.7	0.17	0.0	5.4	0.49	0.0	0.02	5.1
2-F	0.0	0.67	3.3	16	0.0	0.17	3.4	15
3-F	0.0	0.24	1.9	11	0.79	0.0	2.5	12
4-F	0.0	0.30	2.0	8.5	0.0	0.96	2.2	8.7
5-F	0.85	0.0	1.5	8.5	0.70	0.0	0.50	8.6
6-F	0.82	0.0	0.40	5.9	0.51	0.06	0.0	6.4
2-CH_3	0.0	0.24	1.3	6.7	0.0	0.81	2.4	6.9
3-CH_3	0.0	0.24	1.2	7.6	0.0	0.83	2.5	9.0
4-CH_3	0.0	0.43	1.6	7.4	0.0	1.4	2.1	6.9
5-CH_3	0.35	0.0	1.3	7.8	0.0	0.05	1.5	7.9
6-CH_3	0.45	0.0	0.84	6.8	0.0	0.40	1.5	6.9

* NH_2 group on positions 2, 4 and 5 opens possibility for additional tautomers **EN**, **KEN**, **KKN** and **KN** (see the scheme below in the case of 4-substituted compound as an example). The relative energies are given in brackets below the values of the corresponding major tautomers in the table.

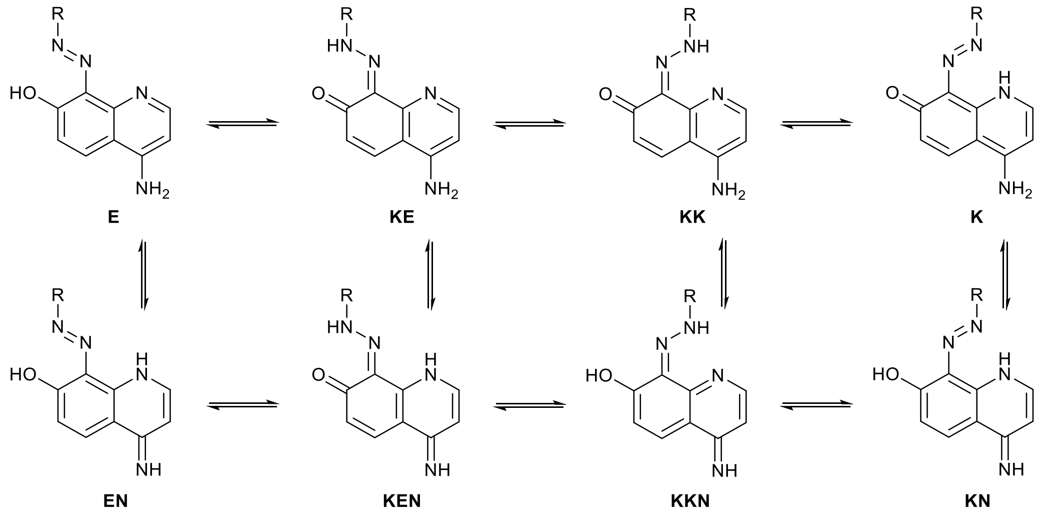
E
KE
KK
K
EN
KEN
KKN
KN
